# Surface Charge Modulation in Covalent Organic Frameworks for Controlled Pt‐Photodeposition and Enhanced Photocatalytic Hydrogen Evolution

**DOI:** 10.1002/smll.202500870

**Published:** 2025-05-19

**Authors:** Klaudija Paliušytė, Lucas Leão Nascimento, Hannah Illner, Max Wiedmaier, Roman Guntermann, Markus Döblinger, Thomas Bein, Antonio Otavio T. Patrocinio, Jenny Schneider

**Affiliations:** ^1^ Department of Chemistry and Center for Nanoscience (CeNS) Ludwig‐Maximilians‐Universität (LMU) Butenandtstraße 11 (E) 81377 Munich Germany; ^2^ Laboratory of Photochemistry and Materials Science (LAFOT‐CM) Institute of Chemistry Federal University of Uberlandia (UFU) Av. João Naves de Ávila Uberlandia 2121 38400‐902 Brazil; ^3^ Centro de Excelência em Hidrogênio e Tecnologias Energéticas Sustentáveis – CEHTES Parque Tecnológico Samambaia Goiânia GO 74690‐631 Brazil

**Keywords:** covalent organic frameworks, hydrogen evolution, photocatalysis, protonation, surface charge

## Abstract

Covalent organic frameworks (COFs) represent a new class of organic photocatalysts for the hydrogen evolution reaction (HER). While the influence of COF structural and optoelectronic properties on HER is well‐studied, the role of surface charge in optimizing interfacial interactions with reactants remains underexplored. In this study, it is demonstrated that converting imine to amide linkages in a thiophene‐based COF allows for altering surface charge through different protonation behaviors of the linkages. Zeta potential measurements reveal that the amide‐linked COF, due to its lower basicity, is deprotonated and negatively charged in the presence of ascorbic acid, while the imine‐linked COF is protonated and positively charged. This electrostatic contrast drives the photoreduction of [PtCl_6_]^2^⁻ to Pt, with the imine‐linked COF yielding uniformly distributed small Pt particles (1–2 nm), whereas the amide‐linked COF forms larger Pt particles (up to 100 nm). The amide‐linked COF, acting as an antenna that facilitates interdomain electron transport along COF agglomerates, promotes both Pt growth and subsequent proton reduction demonstrating a 300% increase in photocatalytic HER rate compared to its imine form. This work introduces surface charge modulation as a novel tool for controlling photocatalytic processes in COF‐based systems expanding the COF functionality in photocatalysis.

## Graphical abstract

This study demonstrates that the photocatalytic hydrogen evolution efficiency of COFs can be effectively controlled by modulating surface charge via post‐synthetic linkage conversion from imine to amide. This unique interfacial modification approach switches the surface charge of COFs and introduces a shift in the reaction mechanism of the in situ Pt‐photodeposition, significantly enhancing H₂ evolution efficiency.

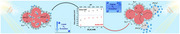

## 1 Introduction

Covalent Organic Frameworks (COFs) have garnered significant attention as promising photocatalysts for H₂ evolution, owing to their crystalline porous structure, high chemical stability, and virtually limitless tunability in terms of both structure and optoelectronic properties.^[^ ^1^ ^]^ In 2014, B. Lotsch et al. reported the first water‐stable hydrazine‐based COF for the hydrogen evolution reaction (HER), achieving a reaction rate of 1979.0 µmol g⁻¹ h⁻¹.^[^ ^2^ ^]^ Subsequent studies have focused on the molecular engineering of COFs to enhance their photocatalytic H₂ evolution efficiency by optimizing light‐harvesting capabilities, charge‐carrier separation, thermodynamic driving force, water dispersibility, and photostability.^[^ ^3^, ^4^, ^5^, ^6^, ^7^, ^8^, ^9^, ^10^, ^11^, ^12^, ^13^ ^]^

Linkage chemistry has proven to be an effective tool for tailoring the physicochemical properties of COFs for photocatalytic applications. X. Liu et al. demonstrated that fully π‐conjugated sp^2^ carbon linkages not only broaden the visible‐light absorption of COFs but also enhance charge transfer and separation efficiency, compared to imine linkages.^[^ ^14^ ^]^ Recently, Thomas et al. showed that protonation of a series of imine‐linked donor‐acceptor COFs enhances photocatalytic H₂ evolution performance, which was attributed to improved light absorption, charge carrier separation efficiency, and hydrophilicity of the COFs upon protonation.^[^ ^15^ ^]^ In a further study, X. Pan and co‐workers investigated the effect of linkage isomerism on photocatalytic hydrogen evolution.^[^ ^16^ ^]^ While the influence of linkages on optoelectronic properties has been widely explored, leveraging COF linkage chemistry to modulate interfacial chemical and electrostatic interactions between COFs and reactants for improved photocatalytic H₂ evolution has yet to be reported.

Typical reactants in the photocatalytic H₂ evolution reaction include charged precursors for co‐catalyst deposition, such as H_2_PtCl_6_, and sacrificial electron donors, which often also serve as proton sources.^[^ ^17^, ^18^ ^]^ Tuning the surface charge to promote the preferential adsorption of specific compounds at the photocatalyst surface, thereby controlling the photocatalytic reaction mechanism, is a well‐established strategy in traditional inorganic photocatalysis.^[^ ^19^ ^]^ This method relies on modulating the electrostatic interactions between the adsorbate and the adsorbent, either by altering the chemical environment or by tailoring the surface charge of the photocatalyst. However, systematic studies on this approach applied to COFs remain rare.

The present study explores and demonstrates the effect of different protonation behavior of imine‐and amide‐linked COFs in acidic conditions on the COF's surface charge and photocatalytic HER. The imine‐linked COF was synthesized from benzo[1,2‐b:4,5‐b′]‐dithiophene‐2,6‐dicarboxaldehyde (BDT) and 4‐fold amine functionalized tetraphenylethylene (1,1′,2,2′‐tetra‐p‐aminophenylethylene) (ETTA) building blocks under solvothermal conditions.^[^ ^20^ ^]^ A post‐modification method was applied to induce imine‐to‐amide linkage conversion.^[^ ^21^ ^]^ This approach resulted in a crystalline Amide‐BDT‐ETTA COF, which demonstrated a 300% increase in photocatalytic HER rate compared to its imine form. Zeta potential measurements of the suspensions of the COFs in the presence of ascorbic acid serving as an electron donor reveal a switch from positive to negative surface charge of the COF upon imine‐to‐amide linkage conversion. The influence of the surface charge on the mechanism of the in situ Pt‐photodeposition and subsequent proton reduction will be discussed among the observed changes in hydrophilicity, and optoelectronic properties.

## 2 Results and Discussion

### 2.1 Structural and Morphological Characterization

**Figure 1:**
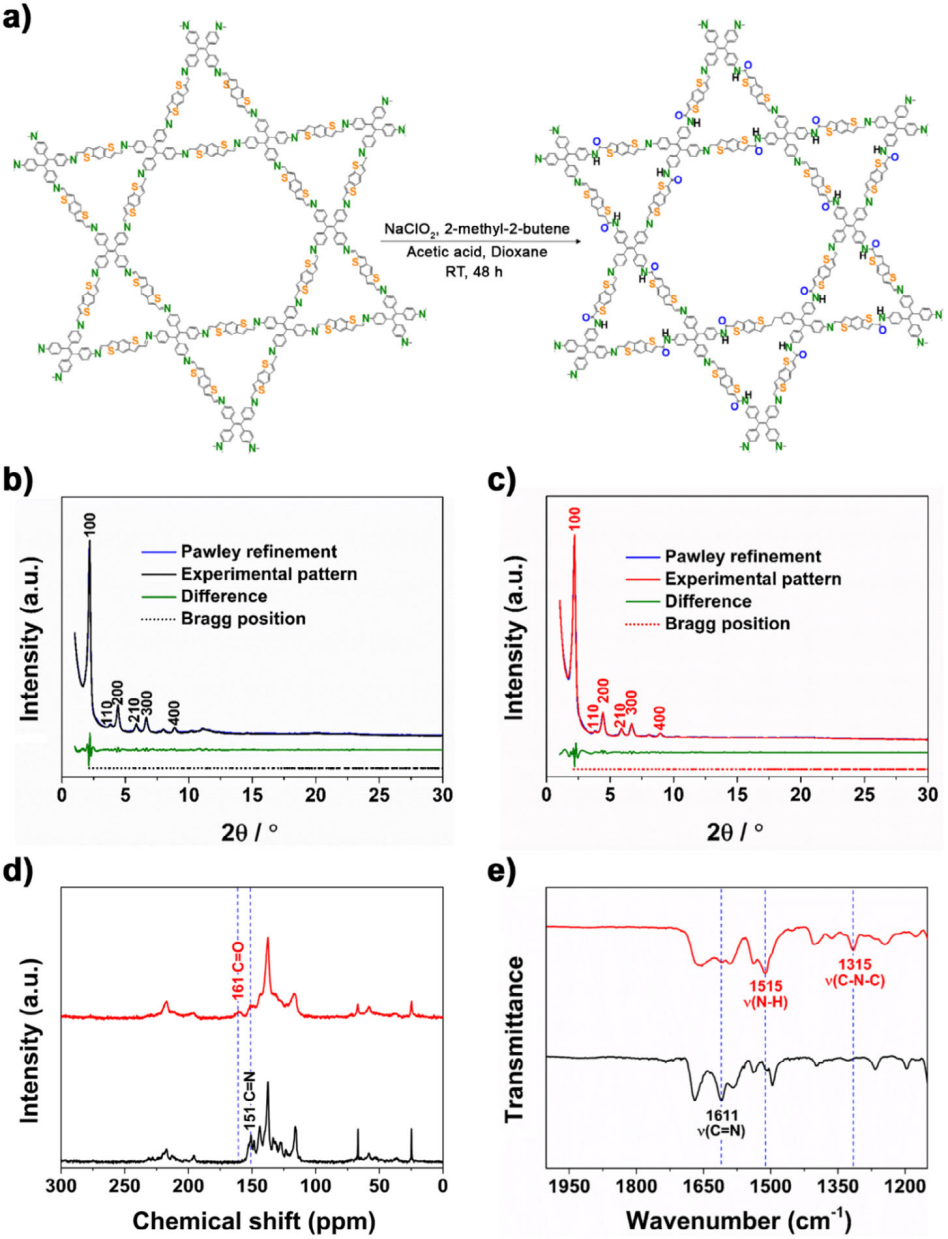
a) Schematic representation of Imine‐BDT‐ETTA to Amide‐BDT‐ETTA conversion. Experimental and Pawley refined PXRD patterns of b) Imine‐BDT‐ETTA and c) Amide‐BDT‐ETTA. d) ^13^C NMR spectra and e) FT‐IR spectra of Imine‐BDT‐ETTA (black) and Amide‐BDT‐ETTA(red).

Imine‐BDT‐ETTA COF was synthesized according to the previously reported procedure using an acceptor‐type ETTA building block and a donor‐type BDT linker.^[^ ^20^ ^]^ The obtained Imine‐BDT‐ETTA was subsequently post‐modified to convert imine to amide linkages using Pinick oxidation reaction^[^ ^22^ ^]^ where sodium chlorite is used as an oxidizing agent, 2‐methyl‐2‐butene as a free radical scavenger, and acetic acid as a buffer during the oxidation process (**Figure** **1**a). The powder X‐ray diffraction (PXRD) patterns shown in Figure 1b for Imine‐BDT‐ETTA COF and in Figure 1c for Amide‐BDT‐ETTA confirm successful synthesis and post‐modification of the COF revealing high crystallinity through the presence of a pronounced and sharp 100 reflection along with well‐defined higher‐order reflections. The structures of Imine‐BDT‐ETTA and Amide‐BDT‐ETTA were simulated using force‐field methods^[^ ^23^ ^]^ comprising a dual‐pore Kagome structure in symmetry (Space Group No. 168) (see Figures S1 and S2, Tables S1 and S2, Supporting Information) with the unit cell parameters being *a* = *b* = 4.64 nm, *c* = 0.446 nm (*Rwp* = 4.6%, *Rp* = 3.7%) and *a* = *b* = 4.78 nm, *c* = 0.448 nm (*Rwp* = 5.7%, *Rp* = 3.8%), respectively. The increase of *a*, *b*, and *c* parameters for amide‐linked COF in comparison to those for the imine counterpart can be attributed to the change of the bond length resulting from the conversion of the C═N double bond (0.129 nm from structural simulations) to the C─N single bond (0.135 nm from structural simulations). For comparison, the structure of Amide‐BDT‐ETTA was also simulated assuming 50% conversion (see Table S3, Supporting Information). The simulated and experimental PXRD patterns were consistent (*Rwp* = 7.8%, *Rp* = 5.2%), demonstrating that regardless of conversion degree to amide bonds, there are no significant changes in the structure of the COF.

Nitrogen physisorption isotherms were used to monitor the changes in the porosity of the COF after the linkage conversion. Like the imine counterpart (Figure S3a, Supporting Information), Amide‐BDT‐ETTA exhibits a sorption isotherm of type IV with two steep nitrogen uptake steps (Figure S3b, Supporting Information). The Brunauer–Emmett–Teller (BET) surface area of Amide‐BDT‐ETTA decreased to 688 m^2^ g^−1^ in comparison to Imine‐BDT‐ETTA (1679 m^2^ g^−1^). Previous studies have reported a decrease in the surface area as a result of post‐synthetic modifications.^[^ ^21^, ^24^, ^25^ ^]^ However, the linkage conversion of the Imine‐BDT‐ETTA did not induce substantial changes in the pore sizes. Using the QSDFT (Quenched Solid Density Functional Theory) method with carbon kernel for cylindrical pores, pore sizes for Imine‐BDT‐ETTA were calculated to be 1.79 and 3.68 nm (vs 1.72 and 3.58 nm based on structural simulations (Figure S1, Supporting Information) aligning well with previous reports.^[^ ^26^ ^]^ Pore sizes for Amide‐BDT‐ETTA were found to be 1.79 and 3.55 nm (1.72 and 3.58 nm from structural simulations Figure S2, Supporting Information).

The morphological characteristics of both COFs were further investigated via scanning electron microscopy (SEM) (Figure S4, Supporting Information). SEM images suggest that Imine‐BDT‐ETTA features a spherical morphology constructed from particles organized in a rose‐shaped agglomerate. Notably, Amide‐BDT‐ETTA shows a more mixed morphology, featuring rod‐like agglomerates alongside remaining rose‐shaped particles. The chemical conversion of the imine‐COF involves a series of processes, including ultrasonication, treatment with different solvents (dioxane, 2‐methyl‐2‐butene), utilization of the inorganic oxidant NaClO_2,_ and exposure to glacial acetic acid which serves as a buffer. To identify the origin of the morphological changes, the following control experiments were conducted: Reference‐COF‐1 was obtained in the absence of NaClO_2_, Reference‐COF‐2 with a fivefold reduction in NaClO_2_ concentration, and Reference‐COF‐3 in the absence of acetic acid, while keeping other ingredients consistent with the original conversion procedure (see the Supporting Information). All three reference samples retained crystallinity after all treatment steps, respectively (Figure S5a, Supporting Information). As expected, Fourier transform infrared (FT‐IR) analysis reveals that for the linkage conversion, the oxidant is required (Figure S5b, Supporting Information). The rod‐shaped particles were observed in all three reference samples (Figure S6, Supporting Information), thus indicating that the formation of such structures is not related to the amidization process itself but rather the result of the ultrasonication and the treatment with organic solvents.

^13^C cross‐polarization magic angle spinning (CPMAS) analysis allowed us to determine the local chemical changes of the COF after linkage conversion (Figure 1d). In the ^13^C NMR spectrum of Amide‐BDT‐ETTA, a distinctive peak at 161 ppm appeared, which is not present in the NMR spectrum of Imine‐BDT‐ETTA prior to the oxidation. This new signal arises from the formation of amide carbonyl C═O moieties.^[^ ^21^ ^]^ Additionally, a residual signal attributed to C═N bonds at 151 ppm remains detectable in Amide‐BDT‐ETTA. This observation implies partial oxidation and suggests that the final structural configuration of Amide‐BDT‐ETTA includes both newly introduced carbonyl C═O functionalities and unreacted imine C═N bonds from its imine precursor COF. We note that harsher conditions, such as prolonged reaction times and increased amount of oxidative agent to induce a full conversion from imine to amide linkages, resulted in a non‐crystalline sample. As demonstrated in previous studies, exposure to strong oxidizing agents can disrupt the crystalline structure by breaking imine bonds.^[^ ^27^ ^]^

FT‐IR spectroscopy was applied to further explore the chemical changes induced through linkage conversion (Figure 1e). Imine‐BDT‐ETTA exhibits a prominent band at 1611 cm^−1^ corresponding to the stretching vibrations of the C═N functional group.^[^ ^28^, ^29^ ^]^ Observed vibration peaks in the FT‐IR spectra of Imine‐BDT‐ETTA are in good agreement with the previously published results.^[^ ^20^, ^30^ ^]^ The emergence of a new band at 1315 cm^−1^ in the Amide‐BDT‐ETTA spectrum reveals the formation of the C–N–C moiety, indicative of the amide linkage.^[^ ^31^ ^]^ Another pronounced band at 1515 cm^−1^ can be attributed to the in‐plane N–H bending vibrations of the secondary amide group.^[^ ^32^, ^33^ ^]^ As previously substantiated by ^13^C solid‐state NMR analysis, the presence of imine bonds is evident in the FT‐IR spectrum of Amide‐BDT‐ETTA as well. Typically, the stretching vibrations of imine and amide bonds are observed in the spectral range of 1600–1690 cm^−1^.^[^ ^21^, ^28^, ^29^, ^34^, ^35^, ^36^, ^37^, ^38^ ^]^ However, within the spectral range of Amide‐BDT‐ETTA, there are multiple overlapping signals, which require more complex analysis. For a deeper analysis of the vibrations associated with the newly formed bonds in the polymeric structure, the FT‐IR spectrum of Imine‐BDT‐ETTA was subtracted from the spectrum of the Amide‐BDT‐ETTA (Figure S7, Supporting Information). In the resulting FT‐IR difference spectrum, the C═O stretching vibration of the amide bond is evident at 1645 cm^−1^.^[^ ^21^, ^32^, ^33^ ^]^

X‐ray photoelectron spectroscopy (XPS) analysis was performed to investigate changes in the oxidation states of the elements present in both COFs (Figure S8, Supporting Information). The survey spectra confirm the absence of impurities, with only peaks corresponding to C, S, O, and N atoms detected. High‐resolution spectra were acquired for each peak and compared to those of the BDT linker (Figure S9, Supporting Information). Focusing initially on the C 1s spectrum of Imine‐BDT‐ETTA (Figure S9a, Supporting Information), the primary peak observed at 284.6 eV is attributed to carbon atoms integrated within the COF framework. A weaker signal at 289.5 eV is linked to residual, unreacted aldehyde groups present at surface terminations and lattice defects. These groups exhibit a higher binding energy due to the reduced electron density surrounding the carbon atoms.^[^ ^39^, ^40^ ^]^ In the case of Amide‐BDT‐ETTA COF, an additional feature appears at 288.1 eV, assigned to the carbonyl (C═O) moiety (Figure S9b, Supporting Information).^[^ ^41^, ^42^ ^]^

In the S 2p region, two distinct sulfur environments are observed in both COFs, characterized by S 2p₃/₂ binding energy peaks at 164.2 and 169.0 eV (Figure S9d,e, Supporting Information). These peaks correspond to neutral sulfur species (S–S, S–C) and oxidized sulfur species (S═O), respectively.^[^ ^43^, ^44^, ^45^ ^]^ Quantitative analysis reveals that Amide‐BDT‐ETTA contains 20% sulfur in the +VI oxidation state, while Imine‐BDT‐ETTA has 15% oxidized sulfur species. Notably, the BDT linker used in the synthesis of Imine‐BDT‐ETTA COF also exhibits 15% oxidized sulfur species (Figure S9f, Supporting Information). These results indicate that COF amidization does not induce significant sulfur oxidation, thereby establishing that the introduction of the amide functionalities is the dominant factor controlling the changes in COF properties.

The primary peak in the O 1s spectra of both COFs (Figure S9g,h, Supporting Information) is attributed to surface‐adsorbed oxygen species.^[^ ^25^ ^]^ In the Amide‐BDT‐ETTA COF, this peak appears at 533.6 eV, shifted relative to that of Imine‐BDT‐ETTA (532.5 eV), indicating differences in surface properties. Additionally, a secondary feature at 531.0 eV is observed for Amide‐BDT‐ETTA, consistent with previously reported values for C═O bonds formed during the amidization reaction.^[^ ^46^ ^]^

In the N 1s region, distinct differences are observed between the two COFs. For Imine‐BDT‐ETTA COF (Figure S9j, Supporting Information), the spectrum can be deconvoluted into two components at binding energies of 398.7 and 402.8 eV, corresponding to imine groups (─C═N─C─) and unreacted amino groups (─C─NH₂) from the ETTA building block, respectively.^[^ ^47^ ^]^ The comparison between the N 1s spectra of Imine‐BDT‐ETTA (Figure S9j, Supporting Information) and Amide‐BDT‐ETTA (Figure S9k, Supporting Information) reveals a shift toward higher binding energy after the amidization process (from 398.7 to 399.2 eV, Figure S9l, Supporting Information). This shift confirms the efficient conversion, consistent with previously reported binding energy changes associated with imine‐to‐amide transformation.^[^ ^25^, ^39^ ^]^

However, the N 1s peak does not allow for direct quantification of imine‐to‐amide conversion yield. Complementary evidence from ¹^3^C─NMR and FTIR confirms the presence of residual imine groups in Amide‐BDT‐ETTA COF. To reliably estimate the conversion yield, we have taken the ratio between the corresponding area for the peaks attributed to the amide C═O (C1s at 288.1 eV and O1s at 531.0 eV) and the N1s in the survey spectra, which should be ideally 1:1. The calculated C═O/N ratio was 0.9, suggesting an approximate conversion yield of 90%.

### 2.2 Photocatalytic Hydrogen Evolution

The as‐prepared imine and amide COFs were tested for photocatalytic hydrogen evolution. The photocatalytic tests were performed in the presence of 1.0 wt% (Pt/COF) H_2_PtCl_6_ precursor and ascorbic acid (H_2_A) acting as sacrificial electron donor (Equations (1), (2), (3), (4)) upon illumination with visible light (λ > 420 nm, 100 mW cm^−2^). After excitation of the COFs, photogenerated electrons (e^−^) and holes (h^+^) are formed (Equation 1). The holes can oxidize H_2_A to dehydroascorbic acid, A, either directly by two‐electron transfer or by one‐electron oxidation via the formation of ascorbyl radical, HA^•^, as an intermediate + H+ (Equations 2 and 3) thus suppressing the undesired recombination. Simultaneously, the photogenerated electrons can reduce 2H^+^ to H_2_ on the surface of in situ formed Pt particles (Equation 4).

(1) $$\mathrm{COF}\overset{hv}{\to }e^{-}+h^{+}\;$$

(2) $$H_{2}A+h^{+}\to HA^{•}+H^{+}$$

(3) $$HA^{•}+h^{+}\to A+H^{+}$$

(4) $$2H^{+}+2e^{-}\to H_{2}$$

**Figure 2:**
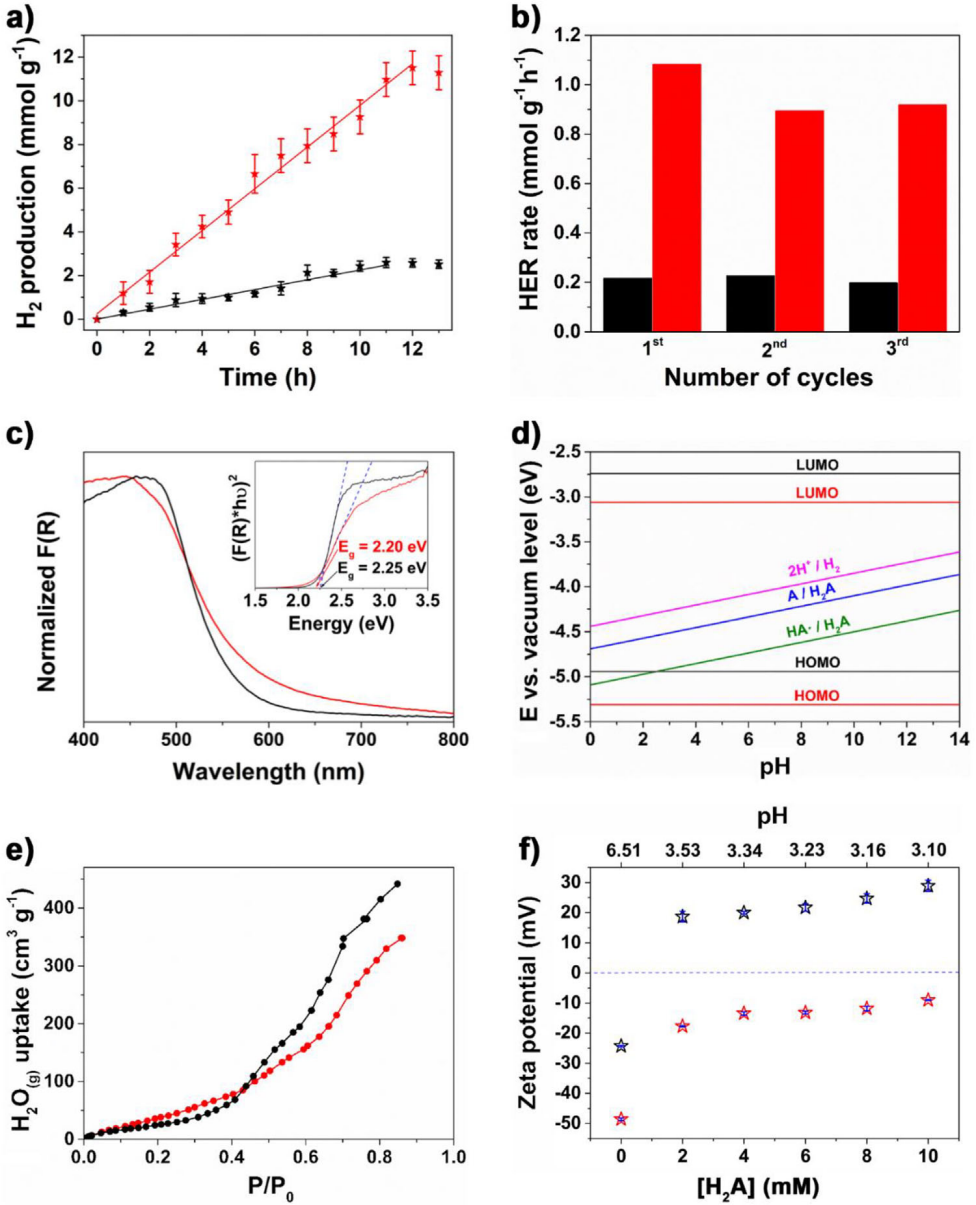
a) Time course of H_2_ evolution and b) hydrogen evolution rates for three cycles, every 13 h obtained with Imine‐BDT‐ETTA (black) and Amide‐BDT‐ETTA (red) including the error bars. Solid lines in (a) represent the linear fitting to extract evolution rates. Conditions of photocatalytic tests: λ > 420 nm, 100 mW cm^−2^, 1 g/l COF suspension containing 10 mM H_2_A and 1.0 wt% (Pt/COF) H_2_PtCl_6_ precursor. c) F(R) and Tauc plots (inset) as well as d) determined energy levels of Imine‐BDT‐ETTA (black) and of Amide‐BDT‐ETTA COF (red) including the redox potentials of the studied photocatalytic reaction. e) Volumetric water vapor adsorption isotherms of Imine‐BDT‐ETTA (black) and Amide‐BDT‐ETTA (red). f) Zeta potential of Imine‐BDT‐ETTA (black) and Amide‐BDT‐ETTA (red) as a function of pH, adjusted using varying concentrations of H₂A (0, 2, 4, 6, 8, and 10 mM) including the error bars (blue).

**Figure** **2**a shows the hydrogen evolution obtained with Imine‐ and Amide‐BDT‐ETTA COFs in the experiments lasting 13 h. From the linear increase of H_2_ production with time, the HER rates for Imine‐BDT‐ETTA and Amide‐BDT‐ETTA were quantified to be 0.22 and 0.95 mmol g^−1^ h^−1^, respectively. These results reveal a pronounced promotion of hydrogen evolution through the linkage conversion. Both COFs were also tested in the absence of light and in the absence of Pt, but no hydrogen evolution was detected. The illumination of the Pt precursor in the presence of ascorbic acid also does not lead to H_2_ evolution. Long‐term photocatalytic experiments were performed in which hydrogen evolution was monitored over three cycles totalizing 39 h (Figure 2b). Both COFs were able to continuously sustain an average HER rate of 0.22 mmol g^−1^ h^−1^ for Imine‐BDT‐ETTA and of 0.95 mmol g^−1^ h^−1^ for Amide‐BDT‐ETTA. In the subsequent three cycles of 3 h each and in the presence of 2 mM H_2_A, even higher evolution rates for both COF were detected, with the Amide‐COF showing an average HER rate of 1.7 mmol g^−1^ h^−1^ while Imine‐BDT‐ETTA delivered 0.40 mmol g^−1^ h^−1^ (Figure S10, Supporting Information).

The HER rate was measured in the presence of various H₂A concentrations using Amide‐BDT‐ETTA as the photocatalyst to investigate the origin of the increased HER rate observed at lower H₂A concentrations. The increase in H_2_A concentration from 2 to 6 mM resulted in a gradual increase of HER rate up to 2.6 mmol g^−1^ h^−1^ (Figure S11a, Supporting Information). Further, increase in the H_2_A concentration from 8 to 10 mM led to a drop in the HER rate. A similar dependence of H_2_ evolution on the concentration of the sacrificial electron donor has been already reported and assigned to a Langmuir‐type catalytic behavior.^[^ ^48^, ^49^, ^50^ ^]^ At low H_2_A concentrations, the HER rate is limited by the mass transfer of the H_2_A to the Pt/COF surface. At high H_2_A concentrations, the coupling of the surface‐adsorbed H^•^ radicals on the Pt surface to form H_2_ gas might be hindered through competing acid adsorption. It has been reported that H_2_A adsorbs at the Pt surface, occupying hydrogen adsorption sites.^[^ ^51^ ^]^ Moreover, H_2_A oxidation is very sensitive to the pH of the reaction media and is favored at higher pH values.^[^ ^52^, ^53^ ^]^ Photocatalytic tests were also conducted using triethanolamine (TEOA) as a sacrificial electron donor (Figure S11b, Supporting Information). Under these alkaline conditions, Imine‐BDT‐ETTA exhibited superior performance compared to Amide‐BDT‐ETTA, with hydrogen evolution rates of 0.63 and 0.39 mmol g⁻¹ h⁻¹, respectively. The change in H₂ evolution rates observed when using ascorbic acid H₂A versus TEOA is typically attributed to differences in the suspension pH, which can influence the adsorption and desorption dynamics and equilibria of reactants and products at the photocatalyst surface.^[^ ^54^, ^55^, ^56^ ^]^ In addition, the decrease in the H_2_ evolution rates for both COFs can be attributed to the higher pH values of the reaction medium (pH = 10.75), thus reducing the availability of protons.^[^ ^57^, ^58^ ^]^

### 2.3 Physical Characterization

To explore the origin of the enhanced H_2_ evolution with Amide‐BDT‐ETTA in comparison to Imine‐BDT‐ETTA, the optoelectronic features of both COFs were examined using cyclic voltammetry (CV), UV‐vis absorption spectroscopy (UV‐vis), and photoluminescence spectroscopy (PL). CV measurements allowed us to quantify the energetic positions of the HOMO levels from half wave potentials for both COFs (Figure S12, Supporting Information). Herein, the experiments were conducted using the Fc/Fc^+^ redox couple as a reference in 0.1 M NBu_4_PF_6_ acetonitrile solutions as described elsewhere.^[^ ^26^ ^]^ The HOMO levels for Imine‐BDT‐ETTA and Amide‐BDT‐ETTA are positioned at −4.94 eV (0.44 V vs SHE) and at −5.31 eV (0.81 V vs SHE), respectively. For a photocatalyst to facilitate photo‐oxidation via photogenerated holes, the HOMO or valence band energy must be more positive (higher potential) than the redox potential of the target oxidation reaction. The more positive HOMO potential of Amide‐BDT‐ETTA compared to its imine counterpart reveals that it is a stronger photooxidizing agent, enhancing its effectiveness in driving photocatalytic oxidation.^[^ ^59^, ^60^ ^]^

The optical properties of the COFs were evaluated by UV‐vis measurements by applying the Kubelka‐Munk function F(R) for solid materials (Figure 2c). In contrast to Imine‐BDT‐ETTA, Amide‐BDT‐ETTA exhibits a strong visible‐light absorption in the spectral region above 510 nm. These findings are consistent with prior studies.^[^ ^61^ ^]^ Assuming direct optical transitions for both polymers, the optical band gap energies were calculated employing the Tauc method, thereby revealing values of 2.25 eV for Imine‐BDT‐ETTA and 2.20 eV for Amide‐BDT‐ETTA.^[^ ^62^, ^63^ ^]^ The stronger optical absorption of the amide‐linked COF above 510 nm might originate from the presence of defect states which could reduce the existing Schottky barrier thus facilitating electron transfer to the Pt and enhancing the proton reduction.^[^ ^64^ ^]^ A Schottky barrier at the semiconducting polymer/metal interface has been reported to result from the large band offset between the electron affinity of the polymer versus the work function of the metal.^[^ ^65^ ^]^ Schottky barriers should impede the electron injection into the metal, however, the presence of surface states in the polymer can dramatically reduce this barrier, thus allowing fast electron injection and enhancing the photocatalytic performance.^[^ ^66^, ^67^ ^]^

The LUMO energy levels for both COFs were calculated from the HOMO energy levels obtained by the CV measurements and the optical bandgap energies. As evident from Figure 2d, the imine to amide linkage conversion has caused a shift in HOMO and LUMO energies to more negative potentials. Xiang et al. reported similar changes upon amidization and attributed the behavior to a more pronounced negative charge accumulation in amide COF as a result of the stronger electron‐withdrawing properties of amide linkages.^[^ ^39^ ^]^ PL spectra of the COFs were recorded by exciting the materials at 375 nm. Imine‐BDT‐ETTA and Amide‐BDT‐ETTA exhibit strong emission bands centered at 617 nm and 608 nm, respectively (Figure S13, Supporting Information). The blue shift for the latter can be explained by the weakened conjugation arising from the oxidation of the imine bonds.^[^ ^68^, ^69^ ^]^

Figure 2d shows the band energy levels for both COFs compared to the redox potentials of the studied photocatalytic reactions. Figure 2d reveals the low thermodynamic driving force for the Imine‐BDT‐ETTA to induce the one‐electron oxidation of H_2_A in the pH range of photocatalytic experiments (from 3.10 to 3.53 as shown in Table S4, Supporting Information). On the other hand, Amide‐BDT‐ETTA can efficiently initiate one and two‐electron oxidation of the H_2_A, thus enabling more efficient electron accumulation required for the targeted H_2_ evolution. Additionally, both COFs are thermodynamically suitable to promote the hydrogen evolution reaction, 2H^+^/H_2_ (Equation 4). Here, the potential variation of the HOMO and LUMO levels of the COFs as a function of the pH was neglected based on prior studies.^[^ ^26^ ^]^ Moreover, the difference in the thermodynamic driving force for proton reduction between Imine‐ and Amide‐BDT‐ETTA (see Figure 2d) is less relevant as no H_2_ was generated in the absence of Pt. This evidences that the proton reduction proceeds via electron transfer at the metal surface.

### 2.4 Interfacial Characterization

Among the thermodynamic driving force for the photocatalytic reaction the interactions at the interface between reactants/products and the photocatalyst is essential. In the photocatalytic hydrogen evolution reaction in aqueous media, the hydrophilic character of the COF might influence the efficiency of the process. Herein, volumetric water sorption experiments were conducted for both COFs. Figure 2e shows that at lower relative pressures (P/P_0_ < 0.45) Amide‐BDT‐ETTA has a greater water vapor sorption in comparison to Imine‐BDT‐ETTA, thus evincing its higher affinity for adsorbate^[^ ^70^, ^71^, ^72^ ^]^ and stronger hydrophilic character. This most likely arises from the presence of the carbonyl groups of the amide.^[^ ^73^ ^]^ The total water vapor uptake for Amide‐BDT‐ETTA was 348 cm^3^ g^−1^ while for Imine‐BDT‐ETTA a value of 443 cm^3^ g^−1^ was found. For an adequate comparison, the previously determined BET surface area has to be considered (Figure S3, Supporting Information). Accordingly, the water vapor uptake normalized to the BET surface for Amide‐BDT‐ETTA is higher (0.51 cm^3^ m^−2^) in comparison to Imine‐BDT‐ETTA (0.26 cm^3^ m^−2^). Hence, the inherent hydrophilic characteristics and increased polarity arising from the presence of C═O bonds in the Amide‐BDT‐ETTA contribute to the elevated HER rate and support the formation of stable suspensions in aqueous media in contrast to Imine‐BDT‐ETTA (Figure S14, Supporting Information).^[^ ^74^ ^]^

The observed HER rate dependence on the H_2_A concentration for Imine‐BDT‐ETTA (see Figure S10a, Supporting Information) and Amide‐BDT‐ETTA (see Figure S10b, Supporting Information) underscores the effect of the COF structure on the hole‐driven H_2_A oxidation, aiming at the promotion of H_2_ evolution. The efficiency of the H_2_A oxidation depends on the thermodynamic driving force (discussed above) and its adsorption at the COF surface. In the studied pH region, H_2_A is present in a protonated form and it can interact with both COFs only through weak H‐bonding formed either with the imine linkage or the C═O bond of amide linkage. Consequently, no adsorption of H_2_A onto the surface of either COFs was detected in the dark (see Figure S15, Supporting Information). However, Amide‐BDT‐ETTA has a greater tendency to form stronger H‐bonds with water molecules, thereby potentially aiding in the eventual reduction of protons.^[^ ^75^ ^]^

The interfacial interactions between the COF and the H₂PtCl₆ precursor present mainly as [PtCl_6_]^2−^ are crucial for photocatalytic H₂ evolution, as they dictate Pt growth and distribution on the COF surface. The coordinating sites for metal complexes in the COF structures have been reported to enable the controlled growth of Pt particles.^[^ ^76^ ^]^ However, the formation of the coordinative bonds may be slower than the charge transfer to the transition metal complexes as the latter is known to happen on nanoseconds to picoseconds timescale.^[^ ^77^ ^]^ Additionally, coordinative binding often requires thermal treatment.^[^ ^78^ ^]^ Hence, interactions such as electrostatic attraction and repulsion between charged particles are most likely to govern the in situ photodeposition of Pt.

For studies of the columbic interactions between the COFs and [PtCl_6_]^2−^ precursor, the surface charge of both COFs was determined by measuring the zeta potential in water‐based suspensions (0.1 g L^−1^) with different H_2_A concentrations (0 to 10 mM). As shown in Figure 2f, both COFs have negative surface charge in the absence of H_2_A. Imine‐BDT‐ETTA ([H_2_A] = 0 mM) shows a zeta potential of −24.4 ± 0.3 mV, while the zeta potential value for Amide‐BDT‐ETTA is −48.6 ± 0.5 mV. The Amide‐BDT‐ETTA exhibits more negative zeta potential (below ‐30 mV), indicating improved stability in aqueous suspensions compared to its imine counterpart.^[^ ^79^ ^]^ The addition of H_2_A shifted the zeta potential of Imine‐BDT‐ETTA to positive values. As shown in previous studies,^[^ ^80^, ^81^ ^]^ such a shift can be caused by the protonation of functional groups. Imine bonds are known to act as weak bases and are capable of being protonated.^[^ ^15^, ^16^ ^]^ In order to calculate the ratio between protonated and non‐protonated forms of the COFs, the Henderson‐Hasselbalch equation for weak bases was used (Equation 5).^[^ ^82^ ^,^ ^83^ ^]^

(5) $$\mathrm{pOH}\;=pK_{b}+\log\frac{\left[AH^{+}\right]}{\left[A\right]}$$

(p*K*_b_: base dissociation constant; [A]: concentration of the base; [AH^+^]: concentration of conjugate acid)

Here p*K_b_* values for imine bonds range between 8 to 10, and their conjugate acids (protonated imines) have p*K_a_* (acid dissociation constant) values between 4 and 6 (because p*K_b_* + p*K_a_* = 14),^[^ ^84^ ^]^ suggesting that protonation of imine linkages occurs at pH levels below 6.^[^ ^85^, ^86^ ^]^ During photocatalytic tests in the presence of H_2_A an acidic environment is achieved (pH = 3.10–3.53), i.e., Imine‐BDT‐ETTA‐COF is predominantly present in protonated form ([A] < [AH^+^]). The positive shift of the zeta potential was also observed for Amide‐BDT‐ETTA, however, it remained in the negative range. The positive shift can be attributed to the protonation of unreacted imine bonds while amide bonds remain non‐protonated due to their lower basicity (p*K_b_* values ranging between 13 and 16 while p*K_a_* for conjugate acids (protonated amides) varies between −2 to 1).^[^ ^87^, ^88^, ^89^, ^90^ ^]^ This aligns well with the well‐known fact that more acidic functional groups typically exhibit more negative zeta potential than the more basic functional groups.^[^ ^91^ ^]^ The protonation of amide linkages occurs in the presence of strong acids at pH values below 1. In the experimental conditions employed for the photocatalytic tests, the amide linkages are in the non‐protonated form ([A] > [AH^+^]) causing negative zeta potential and negatively charged COF particles. These results suggest the electrostatic attraction of negatively charged Pt‐precursor [PtCl_6_]^2‐^ with positively charged Imine‐BDT‐ETTA and electrostatic repulsion with negatively charged Amide‐BDT‐ETTA. Further analysis requires post‐characterization of both COFs.

### 2.5 Post‐Characterization and Proposed Reaction Mechanism

**Figure 3:**
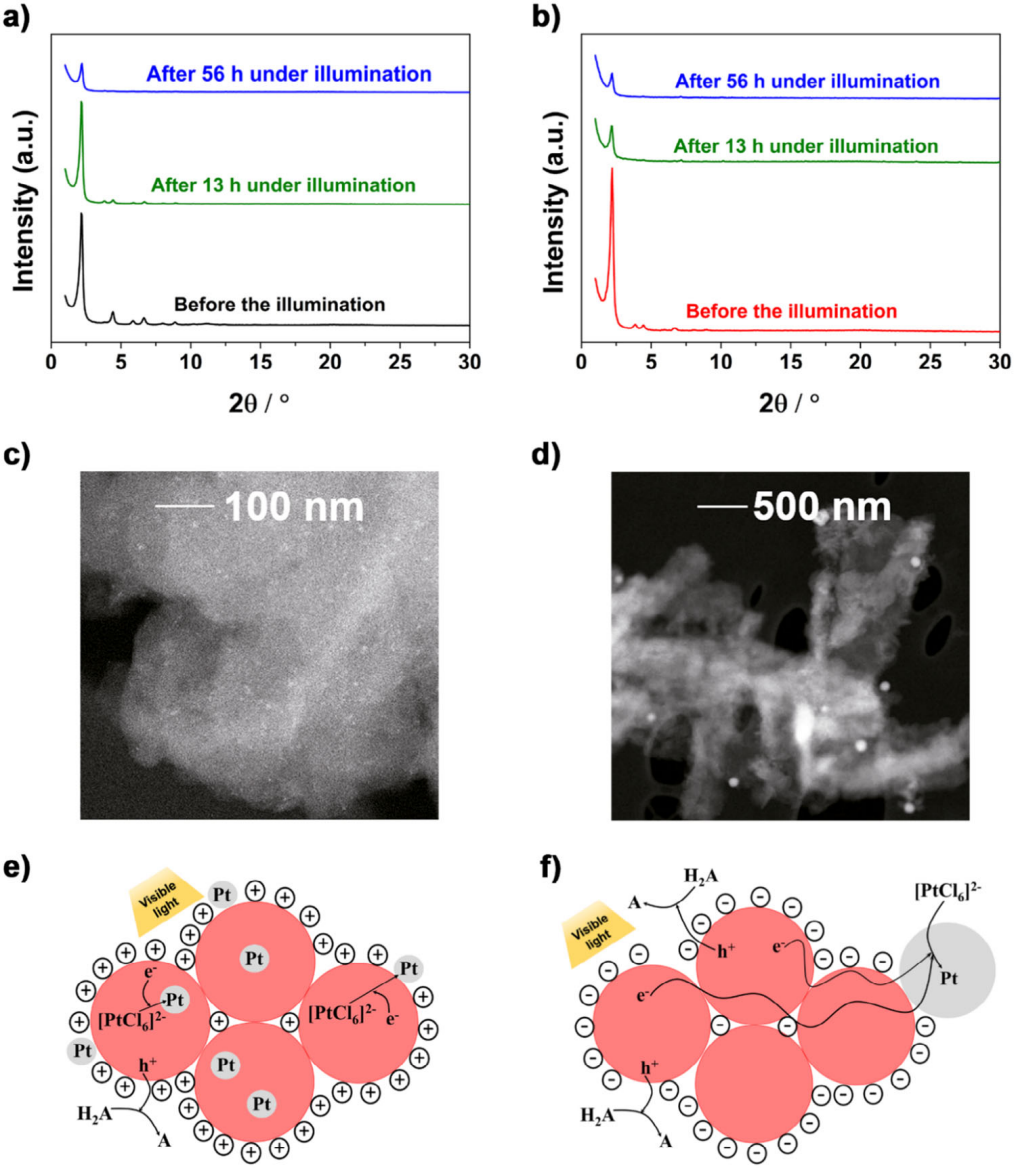
PXRD patterns of a) Imine‐BDT‐ETTA and b) Amide‐BDT‐ETTA before and after 13 and 56 h of photocatalytic test. STEM‐HAADF images showing the distribution of Pt particles in the polymeric structure of c) Imine‐BDT‐ETTA and d) Amide‐BDT‐ETTA after 13 h of photocatalytic test. Conditions of photocatalytic test: λ > 420 nm, 100 mW cm^−2^, 1 g/l COF suspension containing 10 mM H_2_A and 1.0 wt% (Pt/COF) H_2_PtCl_6_ precursor. Schematic representation of interparticle electron transport along agglomerates of e) Imine‐BDT‐ETTA and f) Amide‐BDT‐ETTA toward the Pt particles (red: COF domains, grey: Pt particles).

The consistent and stable rates of hydrogen production observed during the illumination lasting 48 h reveal fair photochemical stability of both COFs. Accordingly, structural characterization of both COFs was conducted after 13 and 56 h of illumination in the presence of 10 mM H_2_A and 1 wt% Pt loading. The PXRD patterns presented in **Figure** **3**a,b reveal that after 13 and 56 h of illumination, both COFs exhibit retention of crystalline structure.

To gain insights into the structural distribution of Pt particles, scanning transmission electron microscopy (STEM) analysis was performed for both COFs following 13 h photocatalytic test. Figure 3c and Figure S16a,b (Supporting Information) reveal a random distribution of small Pt particles dispersed within the matrix of Imine‐BDT‐ETTA. Statistical analysis of the Pt size distribution, Figure S17a (Supporting Information), establishes an average Pt particle size of 3.9 nm with a full width at half maximum (FWHM) of the distribution of 3.6 nm. In contrast, Amide‐BDT‐ETTA (Figure 3d; Figure S16c,d, Supporting Information) displays large Pt particles with a much broader size distribution. As shown in Figure S17b (Supporting Information), the size of the Pt particles grown on the amide‐linked COF lies within two distinct size ranges, i.e., a broad distribution centered around an average size of 90 nm (FWHM = 35 nm), and a narrower distribution with an average size of 12 nm (FWHM = 4 nm). Statistical analysis reveals that ≈90% of Pt particles on Amide‐BDT‐ETTA are larger than those observed on Imine‐BDT‐ETTA. These findings also correlate with the quantified imine‐to‐amide conversion degree of 90% and strongly suggest that the Pt growth mechanism is influenced by the nature of the COF linkage, which modulates the interaction between the COF framework and the Pt precursor during photodeposition.

The attractive interaction between positively charged Imine‐BDT‐ETTA with the negatively charged Pt‐precursor [PtCl_6_]^2−^ should promote the high Pt nucleation rate at numerous sites and result in a homogeneous distribution of small (1–2 nm) Pt particles in the framework (Figure 3e). In contrast, Amide‐BDT‐ETTA is negatively charged in the acidic environment, leading to repulsion between the COF and the [PtCl_6_]^2−^ anions, which is proposed to cause the formation of larger Pt particles. Hereby, the interparticle electron transport along the BDT‐ETTA agglomerates to pre‐formed metal particles enables the formation of larger particles (Figure 3f).

These results reveal the ability of the COF to serve as an antenna for electron transport. The effect of COF surface charge on Pt particle formation and photocatalytic performance is further supported by experiments conducted in the presence of TEOA as a sacrificial agent. Under these conditions, the medium becomes alkaline, and both COFs are non‐protonated^[^ ^85^, ^86^, ^87^, ^88^, ^89^, ^90^ ^]^ and acquire a negative surface charge. Notably, the Imine‐BDT‐COF outperforms the Amide‐COF in this scenario. These results highlight the critical role of surface charge modulation in controlling the efficiency of photocatalytic hydrogen production. Hence, the linkage conversion allowed for tuning of the surface charges of the COF through different protonation behavior of the imine and amide linkage, which had a direct impact on the growth of the Pt particles on the COF surface. An additional tool for controlling the photocatalytic process was demonstrated.

The influence of the Pt particle size on the hydrogen evolution reaction remains a topic of ongoing debate in the literature.^[^ ^92^, ^93^, ^94^, ^95^ ^]^ In general, the overall photocatalytic performance is governed by a sequence of key steps (omitting here the role of the sacrificial donor): i) light absorption, charge separation, and charge transport within the (COF) photocatalyst to the metal co‐catalyst, ii) interfacial electron transfer between the photocatalyst and the metal co‐catalyst, and iii) proton reduction to form hydrogen atoms on the metal surface, followed by H_2_ formation and desorption. While the first step is primarily determined by the intrinsic electronic properties of the (COF) photocatalyst, the second and third steps are influenced by the nature and distribution of the metal particles, particularly their size and interfacial characteristics, including electronic coupling to the COF. The efficiency of charge separation and interfacial electron transfer can indeed be modulated by the Pt particle size, which plays a critical role in facilitating or hindering these processes.^[^ ^94^, ^96^ ^]^ However, the Volmer step—the initial formation of adsorbed hydrogen atoms (H^•^) on the Pt surface—is the rate‐limiting step in catalytic hydrogen evolution. Bard and co‐workers demonstrated that the kinetics of this step are accelerated as Pt particle size increases on Bi and Pb substrates.^[^ ^97^ ^]^ In light of these findings, we propose that the larger Pt particles formed on Amide‐BDT‐ETTA may enhance H₂ production due to improved kinetics of the Volmer step. However, we emphasize that systematic studies on the relationship between Pt particle size, charge separation efficiency, interfacial electron transfer, and hydrogen formation kinetics in COF‐based systems are still lacking and warrant further investigation.

## 3 Conclusion

This study demonstrates how the structural, optoelectronic, and interfacial properties of the BDT‐ETTA COF are modulated through linkage conversion from imine to amide and their resulting influence on photocatalytic performance. Retaining its crystallinity upon amidization, the amide‐linked COF exhibits a shift in energy levels toward more positive potentials (E vs SCE), enhancing its oxidizing power. This stronger oxidation capability facilitates more efficient hole scavenging, thereby promoting proton reduction while suppressing charge carrier recombination. Additionally, the increased hydrophilicity of Amide‐BDT‐ETTA, attributed to the presence of C═O bonds, leads to an improved HER rate and enhances the stability of its suspensions in aqueous media compared to Imine‐BDT‐ETTA. Notably, our findings reveal, for the first time, that surface charge modulation, driven by the distinct protonation behavior of imine and amide linkages, plays a critical role in photocatalytic performance. This interfacial modification strategy effectively switches the surface charge of COFs and alters the in situ Pt‐photodeposition mechanism. Specifically, the negatively charged Amide‐BDT‐ETTA promotes proton adsorption and facilitates the formation of large Pt particles (up to 100 nm), which are highly effective for proton reduction. By highlighting the impact of interfacial properties on reaction mechanisms and photocatalytic hydrogen evolution, this study expands the functional versatility of COFs in photocatalysis. We anticipate that this approach can be further applied to a wide range of photocatalytic reactions and diverse linkage motifs, offering new pathways for designing advanced COF‐based photocatalysts.

## Conflict of Interest

The authors declare no conflict of interest.

## Supporting information



Supporting Information

## Data Availability

The data that support the findings of this study are available from the corresponding author upon reasonable request.
