# Supplementary material for: Surface Charge Modulation in Covalent Organic Frameworks for Controlled Pt‐Photodeposition and Enhanced Photocatalytic Hydrogen Evolution
Source: Small. 2025 May 19;21(27):2500870. doi: 10.1002/smll.202500870 (PMC12243704; doi:10.1002/smll.202500870)
Supplement: Supplementary file 1 — Supporting Information [file SMLL-21-2500870-s001.docx]

Supporting Information

**Surface Charge Modulation in Covalent Organic Frameworks for Controlled Pt-Photodeposition and Enhanced Photocatalytic Hydrogen Evolution**

*Klaudija Paliušytė,* *Lucas Leão Nascimento, Hannah Illner, Max Wiedmaier, Roman Guntermann, Markus Döblinger, Thomas Bein, Antonio Otavio T. Patrocinio*, and Jenny Schneider**

Klaudija Paliušytė, Hannah Illner, Max Wiedmaier, Roman Guntermann, Dr. Markus Döblinger, Prof. Dr. Thomas Bein, Dr. Jenny Schneider

Department of Chemistry and Center for Nanoscience (CeNS), Ludwig-Maximilians-Universität (LMU), Butenandtstraße 11 (E), 81377 Munich, Germany

E-mail: jenny.schneider@cup.uni-muenchen.de

Lucas Leão Nascimento, Prof. Dr. Antonio Otavio T. Patrocinio

Laboratory of Photochemistry and Materials Science (LAFOT-CM), Institute of Chemistry Federal University of Uberlandia (UFU) Av. João Naves de Ávila, 2121 38400-902 Uberlandia, Brazil

E-mail: otaviopatrocinio@ufu.br

Prof. Dr. Antonio Otavio T. Patrocinio

Centro de Excelência em Hidrogênio e Tecnologias Energéticas Sustentáveis – CEHTES, Parque Tecnológico Samambaia, 74690-631, Goiânia, GO, Brazil

**Experimental section**

**Chemicals.** All materials were purchased from Aldrich, Fluka, Acros, Activate Scientific, or TCI Europe in the common purities purum, puriss, or reagent grade. Materials were used as received without additional purification and handled under air unless noted otherwise. All used solvents were anhydrous and purged with inert gas.

**Benzo[1,2-*b*:4,5-*b*’]dithiophene-2,6-dicarboxaldehyde (BDT).**

2,6-benzo[1,2-b:4,5-b′]dithiophene dialdehyde (BDT) was synthesized as reported in the literature.^[1]^ Benzo[1,2-*b*:4,5-*b*’]dithiophene (1.0 g, 5.3 mmol) was dissolved in 100 mL anhydrous, inhibitor-free THF in an outgassed 250 mL flask under nitrogen atmosphere. The stirred solution was cooled to -78°C in a dry ice/acetone cooling bath. A solution of *n*-butyl lithium (5.0 mL, 2.5 M in *n*-hexane, 12.5 mmol) was added dropwise within 10 minutes. The solution was stirred for 30 minutes at -78°C and after removal of the cooling bath for 1 h at room temperature. The solution was then cooled again to -78°C and DMF (1.0 mL, 13.0 mmol) was added dropwise. After stirring overnight, the solution was poured into ice water and filtered. The yellow precipitate was washed with water several times and further treated with small amounts of MeOH and Et_2_O. The solid was dried under reduced pressure, giving a yellow solid with 46% yield. ^1^H NMR (400 MHz, DMSO-*d6*) δ (ppm): 8.53 (s, 2H), 8.89 (s, 2H), 10.20 (s, 2H); ^13^C NMR (100 MHz, DMSO-*d6*) δ (ppm): 121.6, 135.3, 138.4, 138.9, 144.9, 186.6.

**Imine-BDT-ETTA COF.** Under argon atmosphere, BDT (benzo[1,2-b:4,5- b′]dithiophene-2,6-dicarboxaldehyde, 74 mg, 0.30 mmol) and ETTA (1,1,2,2-tetra(p-aminophenyl)ethylene, 58.6 mg, 0.15 mmol) were suspended in a solvent mixture of benzyl alcohol and mesitylene (v/v 9:1, 5 mL) in a 25 mL Schott-Duran vial. Acetic acid (6 M, 500 μL) was added to the vessel, and the mixture was placed in a preheated oven at 120 °C for 3 days. The resulting orange precipitate was suction filtered, Soxhlet-extracted with dry THF, and dried under reduced pressure, yielding 70 mg of the final material (53% yield).

**Amide-BDT-ETTA COF.** Conversion of BDT-ETTA from imine to amide linkages was adapted from the literature.^[2]^ 10 mg of Imine-BDT-ETTA was dispersed in 10 ml of dioxane and the mixture was ultrasonicated for 30 min to obtain a uniform dispersion. The mixture was then centrifuged to separate the solid and liquid phases, and the excess dioxane was removed to obtain a final volume of 1 ml. To a suspension of Imine-BDT-ETTA (10 mg, 0.025 mmol by imine) in dioxane (1 mL) was added 2-methyl-2-butene (1274 μL, 12.0 mmol, 480 equiv), aqueous sodium chlorite solution (200 μL, 3.3 M, 0.66 mmol, 26.4 equiv), and glacial acetic acid (68.8 μL, 1.2 mmol, 48 equiv) in sequence. The biphasic suspension was let stand without stirring at room temperature in the dark for 48 h, after which an additional portion of sodium chlorite solution (200 μL, 3.3 M, 0.66 mmol, 26.4 equiv) was added. Thereafter, Amide-BDT-ETTA was isolated by filtration and washed with water (10 mL), then 10% sodium thiosulfate (10 mL), then water (10 mL) and finally acetone (10 mL). This resulting powder was Soxhlet-extracted with methanol and dry THF in sequence, followed by drying under reduced vacuum at room temperature for 16 h, yielding 4 mg of the final material (40% yield).

**Reference-COF-1.** 10 mg of Imine-BDT-ETTA was dispersed in 10 ml of dioxane and the mixture was ultrasonicated for 30 min to obtain a uniform dispersion. The mixture was then centrifuged to separate the solid and liquid phases, and the excess dioxane was removed to obtain a final volume of 1 ml. To a final suspension of Imine-BDT-ETTA (10 mg, 0.025 mmol by imine) in dioxane (1 mL) was added 2-methyl-2-butene (1274 μL, 12.0 mmol, 480 equiv), and glacial acetic acid (68.8 μL, 1.2 mmol, 48 equiv) in sequence. The biphasic suspension was let stand without stirring at room temperature in the dark for 48 h, after which Reference-COF-1 was isolated by filtration and washed with water (10 mL), then 10% sodium thiosulfate (10 mL), then water (10 mL) and finally acetone (10 mL). The resulting material was Soxhlet-extracted with methanol and dry THF in sequence, followed by drying under reduced vacuum at room temperature for 16 h.

**Reference-COF-2.** 10 mg of Imine-BDT-ETTA was dispersed in 10 ml of dioxane and the mixture was ultrasonicated for 30 min to obtain a uniform dispersion. The mixture was then centrifuged to separate the solid and liquid phases, and the excess dioxane was removed to obtain a final volume of 1 ml. To a final suspension of Imine-BDT-ETTA (10 mg, 0.025 mmol by imine) in dioxane (1 mL) was added 2-methyl-2-butene (1274 μL, 12.0 mmol, 480 equiv), aqueous sodium chlorite solution (40 μL, 3.3 M, 0.66 mmol, 26.4 equiv), and glacial acetic acid (68.8 μL, 1.2 mmol, 48 equiv) in sequence. The biphasic suspension was let stand without stirring at room temperature in the dark for 48 h, after which Reference-COF-2 was isolated by filtration and washed with water (10 mL), then 10% sodium thiosulfate (10 mL), then water (10 mL) and finally acetone (10 mL). The resulting material was Soxhlet-extracted with methanol and dry THF in sequence, followed by drying under reduced vacuum at room temperature for 16 h.

**Reference-COF-3.** 10 mg of Imine-BDT-ETTA was dispersed in 10 ml of dioxane and the mixture was ultrasonicated for 30 min to obtain a uniform dispersion. The mixture was then centrifuged to separate the solid and liquid phases, and the excess dioxane was removed to obtain a final volume of 1 ml. To a final suspension of Imine-BDT-ETTA (10 mg, 0.025 mmol by imine) in dioxane (1 mL) was added 2-methyl-2-butene (1274 μL, 12.0 mmol, 480 equiv), and aqueous sodium chlorite solution (200 μL, 3.3 M, 0.66 mmol, 26.4 equiv in sequence. The biphasic suspension was let stand without stirring at room temperature in the dark for 48 h, after which Reference-COF-3 was isolated by filtration and washed with water (10 mL), then 10% sodium thiosulfate (10 mL), then water (10 mL) and finally acetone (10 mL). The resulting material was Soxhlet-extracted with methanol and dry THF in sequence, followed by drying under reduced vacuum at room temperature for 16 h.

**Nitrogen sorption measurement.** Nitrogen sorption isotherms were recorded on a Quantachrome Autosorb 1 at 77 K within a pressure range from *P/P_0_* = 0.001 to 0.98. Prior to the measurement of the sorption isotherms, the samples were heated for 24 h at 120 °C under turbo-pumped vacuum. For the evaluation of the surface area the BET model was applied between 0.05 and 0.28 *P/P_0_*. Pore size distributions were calculated using the QSDFT equilibrium model with a carbon kernel for cylindrical pores.

**Powder X-ray diffraction (PXRD)** **measurements.** Powder X-ray diffraction measurements were performed on a Bruker D8 Discover diffractometer using Ni-filtered Cu Kα radiation and a position sensitive LynxEye detector in Bragg-Brentano geometry.

The **structure models of the COFs** were constructed on the basis of the previously reported Imine-BDT-ETTA COF structure^[3]^ using the Accelrys Materials Studio software package. For each COF *P6* symmetry was applied. The structure models were optimized using the Forcite module with the Dreiding force-field. Structure refinements using the Pawley method were carried out as implemented in the Reflex module of the Materials Studio software. Thompson-Cox-Hastings peak profiles were used, and peak asymmetry was corrected using the Berar-Baldinozzi method.

**Solid state ^13^C NMR analysis.** The solid state ^13^C cross-polarization magic angle spinning (CP/MAS) spectra were obtained on a Bruker Avance III-500 solid state NMR spectrometer with a 4 mm double resonance MAS probe and at a MAS rate of 10.0 kHz with a contact time of 2-5 ms and a pulse delay of 4 s.

**Infrared (IR) spectra.** Infrared (IR) spectra were recorded on a Perkin Elmer Spectrum BX II FT-IR system and a Thermo Scientific Nicolet™ 6700 FT-IR spectrometer in transmission mode. IR data are reported in wavenumbers (cm^–1^).

**Ultraviolet–Vis–infrared absorption spectra.** Ultraviolet–Vis–infrared absorption spectra were recorded on a Perkin-Elmer Lambda 1050 spectrometer equipped with a 150 mm integrating sphere.

**Scanning electron microscopy (SEM) images.** SEM images were recorded with an FEI Helios NanoLab G3 UC scanning electron microscope equipped with a field emission gun operated at 3 kV. Prior to the measurements, the samples were sputtered with carbon.

**Transmission electron microscopy (TEM) images.** TEM images were recorded with an FEI Titan Themis 60 - 300 equipped with a field emission gun operated at 300 kV.

**X-ray photoelectron spectroscopy (XPS) measurement.** The XPS measurements were performed with a VSW TA10 X-ray source providing non-monochromatized Al Kα radiation (h*ν* = 1486.6 eV) set at 15 mA and 12 kV and a VSW HA100 hemispherical analyzer. The spectra were recorded with a pass energy of 22 eV and a dwell time of 0.1 s per measurement point. The samples were prepared by drop-casting a dispersion of the respective COF powder in acetonitrile on a silicon wafer. After drying the samples by 60 ºC for 24 h they were transferred to the UHV chamber. The obtained spectra were fitted in Igor Pro 6.0.2.4 using a convolution of Doniach-Ŝunjić and Gaussian functions after a linear background subtraction. For advanced data analysis we applied CasaXPS 2.3.15 processing software based on the survey spectra of the samples.^[4]^

**Photodeposition of Pt and photocatalytic setup.** For each photocatalytic assay, 5 mg of the as-prepared COFs were dispersed in 50 mL of ascorbic acid (H_2_A) aqueous solution with concentration varying from 2 to 10 mM. Following, a proper amount of platinum precursor (H_2_PtCl_6_) was added to the suspension in order to obtain 1.0 wt% of platinum loading. The suspension was sonicated for 30 minutes. A custom-made jacketed borosilicate reactor was used to conduct the photocatalytic reactions, which was kept at 20 ºC through a circulating bath. The reactor was illuminated by a Newport 300 W arc Xe lamp, using a 420 nm LP filter. The irradiation intensity was adjusted to 100 mW cm^-2^ using a Newport 1916-C powermeter equipped with an 818-UV/DB optical detector.

**Photocatalytic H_2_ production tests.** During the H_2_ evolution experiments, aliquots of 500 μL were sampled from the headspace of the reactor, using a gas-tight syringe, and then injected in a PerkinElmer Clarus 580 gas chromatograph equipped with a thermal conductivity detector (GC-TCD). A molecular sieve coupled with a Porapak N column was used to separate the gas products. Argon was used as carrier gas at 10 mL min^-1^ and the TCD filament was kept at 250 ºC.

The generated H₂ was quantified using a calibration curve established by sampling known concentrations of analytical standard H₂ under the same experimental conditions as those used during the photocatalytic assays. Control experiments conducted in the absence of light, platinum precursor, or sacrificial agent showed no H₂ evolution, confirming the necessity of these components for the reaction.

The photonic efficiency of a photocatalytic system is determined by the ratio between the number of reacted molecules and the number of incident photons. It is expressed as follows:


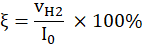


Where ν_H2_ represents the H_2_ evolution rate (mol s^-1^) and I_0_ is the photon flux at 450 nm (einstein s^-1^), the wavelength at which both samples exhibit maximum absorption.

For amide-COF, the highest H_2_ evolution rate obtained was 1.7x10^-3^ mol g^-1^ h^-1^, which corresponds to:


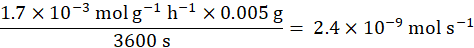


The photon flux at 450 nm, considering a 3.0 cm diameter round-shaped beam with intensity of 100 mW cm^-2^, is approximately 2.67x10^-6^ einstein s^-1^. Thus, the photonic efficiency is calculated as follows:


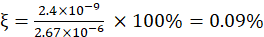
 (Amide-BDT-ETTA COF)

Similarly, for imine-COF, the highest H_2_ evolution rate was 5.6x10^-10^ mol s^-1^, resulting in photonic efficiency of:


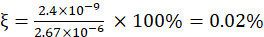
 (Imine-BDT-ETTA COF)

**Cyclic voltammetry (CV) measurements.** COF working electrodes were prepared using an ink made by mixing 5 mg of COF and 3 mg of carbon black in 50 uL of Nafion (∼5 % in a mixture of lower aliphatic alcohols and water) and 450 uL of dimethylformamide. The mixture was then sonicated for 30 minutes. Subsequently, the prepared ink was drop-cast onto the surface of clean FTO glass. The obtained films were dried under vacuum and then used as anodes for the electrochemical experiments. The electrochemical setup was made of a single chamber electrochemical cell, filled with 0.1 M NBu_4_PF_6_ in acetonitrile, using a Pt wire as counter electrode and Ag as pseudo-reference standardized to the ferrocene/ferrocenium couple. The CV measurements were carried out on an Autolab PGSTAT204 potentiostat/galvanostat at 0.1 V s^-1^ scan speed. The pH dependent absolute potential for the HER was calculated as follows:

*E_Abs, pH_* = -4.5 eV + 0.059 · pH

The band edges of the studied COFs were calculated considering that the absolute energy of the Fc/Fc^+^ redox couple is -5.14 eV relative to the vacuum level, as described elsewhere.^[3]^

**Zeta potential measurements.** Zeta potential measurements were recorded using a Malvern Zetasizer instrument at room temperature using 10 mm path length cuvettes by determining the electrophoretic mobility and then applying the Henry equation. The electrophoretic mobility is obtained by performing an electrophoresis experiment on the sample and measuring the velocity of the particles using Laser Doppler

Velocimetry (LDV).

Henry equation:


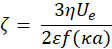


*ζ:*  zeta potential, calculated from electrophoretic mobility (mV),

*η*: viscosity of the medium (in this study 0.8872 mPa s),

*U_e_*: electrophoretic mobility, measured **directly** in a given sample in each round of experiment (m^2^ V^-1^ s^-1^),

*ε*: dielectric constant (in this study 78.5),

*f*(*κa*): Henry's function. According to the Smoluchowski approximation, which is typically used for aqueous samples, *f*(*κa*)  = 1.5.

The samples for the measurement were prepared as follows. 1 mg of the COF was dispersed in 20 ml water following by adding the appropriate amount of H_2_A to achieve 2 to 10 mM in the final suspensions. Measurements were performed in three independent rounds, with each round consisting of 50 measurement cycles.


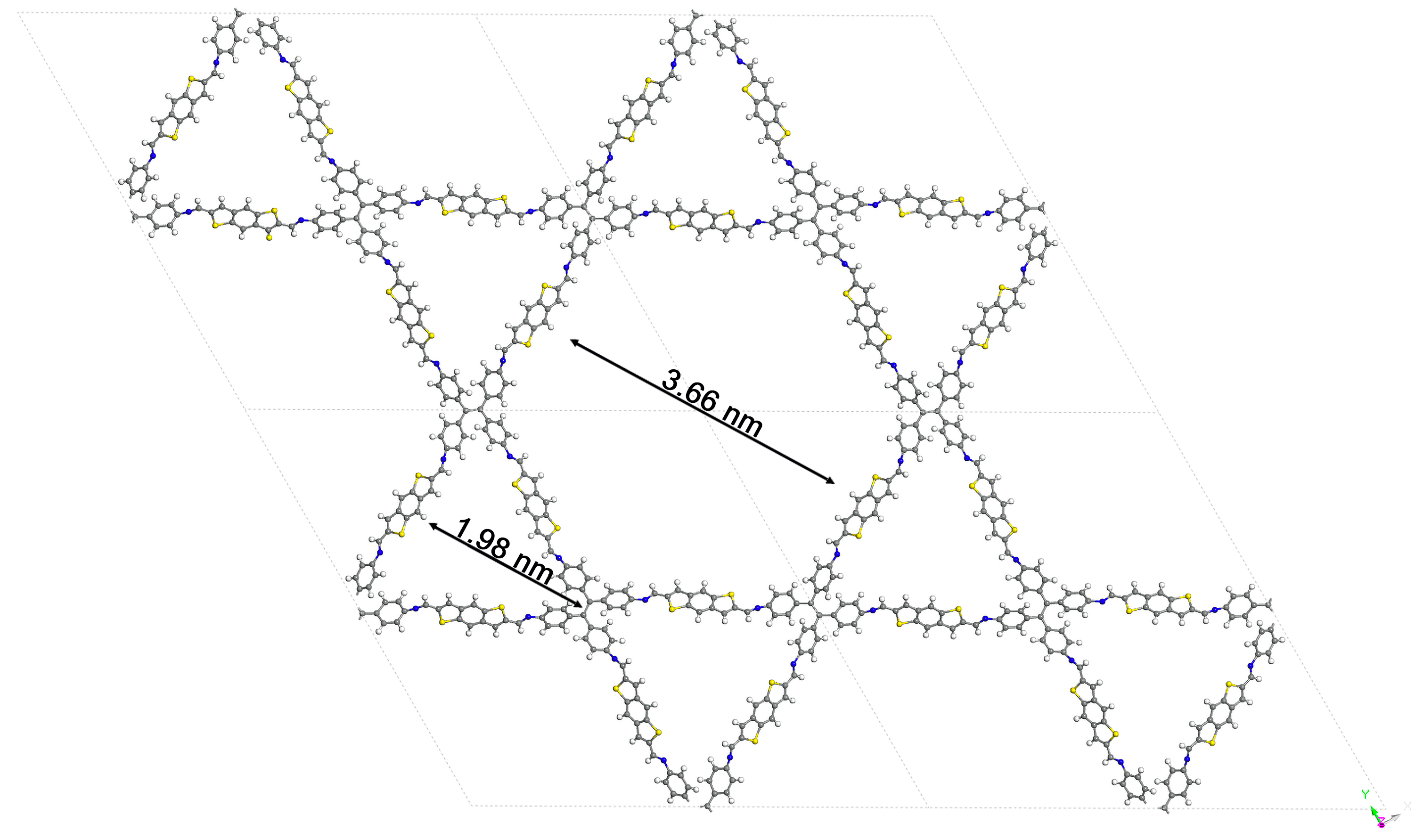


**Figure S1.** Simulated Kagome structure of Imine-BDT-ETTA.

**Structural parameters of Imine-BDT-ETTA:**

Unit Cell Parameters (*P6*):

*a* = *b* = 4.641 nm, *c* = 0.447 nm

*α* = *β* = 90 º, *γ* = 120 º

**Table S1.** Fractional coordinates of Imine-BDT-ETTA.

| C1 | C | 0.48488 | 148.320 | 0.27866 |
| --- | --- | --- | --- | --- |
| C2 | C | 0.51472 | 0.54810 | 0.28876 |
| C3 | C | 0.54703 | 0.52009 | 0.32098 |
| C4 | C | 0.57525 | 0.54268 | 0.15422 |
| C5 | C | 0.60616 | 0.54449 | 0.19731 |
| C6 | C | 0.60923 | 0.52384 | 0.41010 |
| C7 | C | 0.58184 | 0.50322 | 0.58725 |
| C8 | C | 0.55167 | 0.50259 | 0.55024 |
| C9 | C | 0.46020 | 0.42402 | 0.44830 |
| C10 | C | 0.46340 | 0.39632 | 0.52071 |
| C11 | C | 0.49111 | 0.39437 | 0.43094 |
| C12 | C | 0.51510 | 0.41916 | 0.25108 |
| C13 | C | 0.51285 | 0.44705 | 0.17380 |
| N14 | N | 0.50224 | 0.62709 | 0.55696 |
| C15 | C | 0.49283 | 0.64461 | 0.69314 |
| N16 | N | 0.63889 | 0.52197 | 0.45301 |
| C17 | C | 0.66745 | 0.53819 | 0.31775 |
| C18 | C | 0.49687 | 0.32253 | 0.61467 |
| S19 | S | 0.46352 | 0.29767 | 0.34968 |
| C20 | C | 0.47186 | 0.26430 | 0.42403 |
| C21 | C | 0.49747 | 0.27299 | 0.62728 |
| C22 | C | 0.51131 | 0.30666 | 0.73550 |
| C23 | C | 0.50801 | 0.25069 | 0.71007 |
| C24 | C | 0.49177 | 0.21909 | 0.58145 |
| C25 | C | 0.46596 | 0.20993 | 0.37917 |
| C26 | C | 0.45541 | 0.23242 | 0.29525 |
| C27 | C | 0.45299 | 0.17604 | 0.27100 |
| C28 | C | 0.46890 | 0.16145 | 0.39059 |
| S29 | S | 0.50186 | 0.18726 | 0.65424 |
| H30 | H | 0.43913 | 0.42443 | 0.54517 |
| H31 | H | 0.44527 | 0.37716 | 0.66617 |
| H32 | H | 0.53703 | 0.41799 | 0.18828 |
| H33 | H | 0.53437 | 0.46629 | 0.06758 |
| H34 | H | 0.53125 | 0.31766 | 0.89847 |
| H35 | H | 0.52801 | 0.25749 | 0.86879 |
| H36 | H | 0.43562 | 0.22572 | 0.13452 |
| H37 | H | 0.43324 | 0.16431 | 0.10728 |
| H38 | H | 0.44331 | 0.11526 | 0.14900 |
| H39 | H | 0.52604 | 0.36730 | 0.86435 |
| H40 | H | 0.62692 | 0.56104 | 0.05796 |
| H41 | H | 0.57300 | 0.55797 | 0.01601 |
| H42 | H | 0.58421 | 0.48774 | 0.75540 |
| H43 | H | 0.53060 | 0.48739 | 0.68770 |


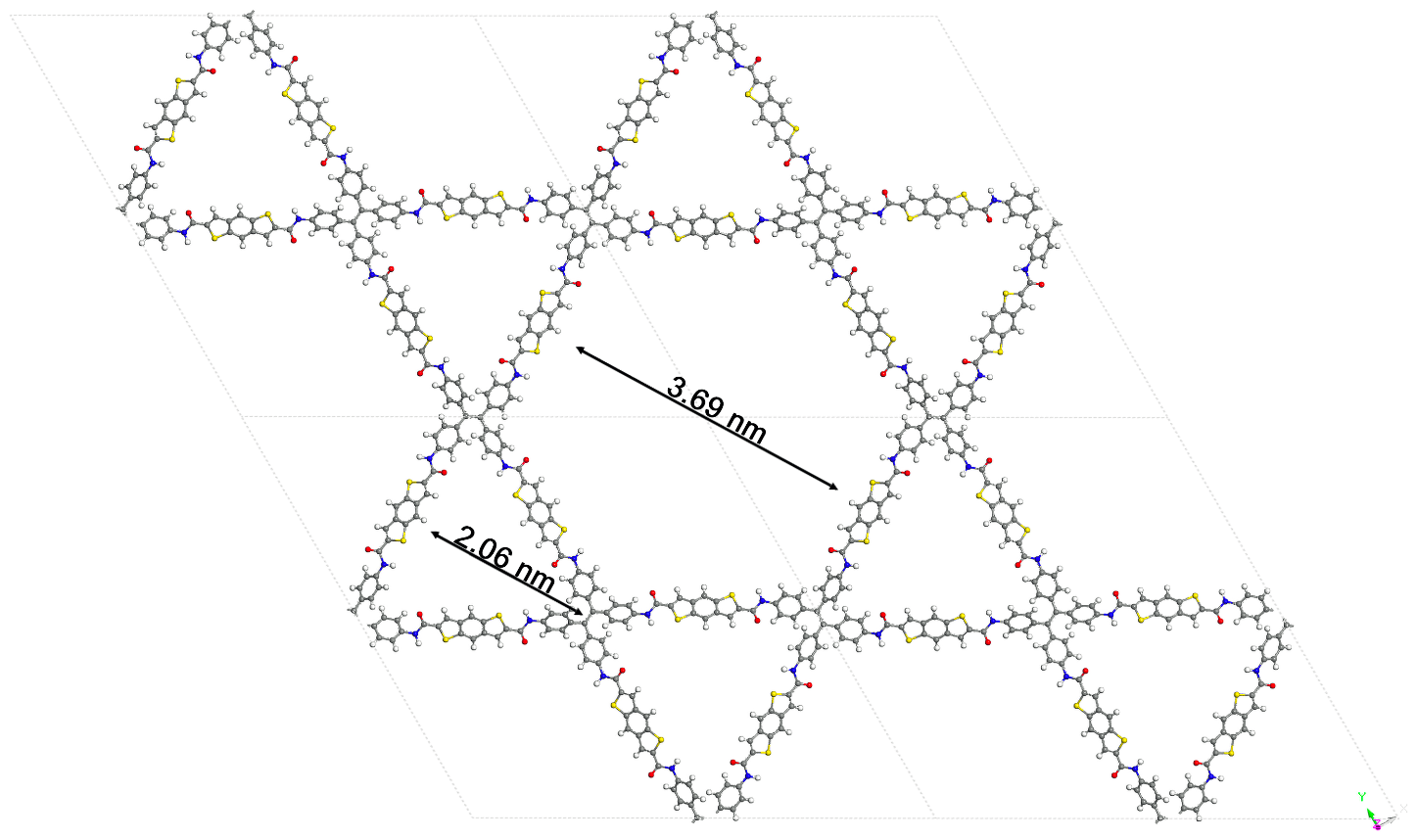


**Figure S2.** Simulated Kagome structure of Amide-BDT-ETTA.

**Structural parameters of fully (100%) amidized Amide-BDT-ETTA**

Unit Cell Parameters (*P6*):

*a* = *b* = 4.781 nm, *c* = 0.448 nm

*α* = *β* = 90 º, *γ* = 120 º

**Table S2.** Fractional coordinates of fully (100%) oxidized Amide-BDT-ETTA.

| C1 | C | 0.48369 | 148.521 | 0.33321 |
| --- | --- | --- | --- | --- |
| C2 | C | 0.52241 | 0.54821 | 0.33528 |
| C3 | C | 0.54474 | 0.51214 | 0.36387 |
| C4 | C | 0.57274 | 0.53115 | 0.18995 |
| C5 | C | 0.60124 | 0.52990 | 0.23550 |
| C6 | C | 0.60253 | 0.50973 | 0.45772 |
| C7 | C | 0.57461 | 0.49027 | 0.62587 |
| C8 | C | 0.54663 | 0.49256 | 0.58555 |
| C9 | C | 0.44904 | 0.42667 | 0.47581 |
| C10 | C | 0.44250 | 0.39460 | 0.49319 |
| C11 | C | 0.46443 | 0.38614 | 0.37885 |
| C12 | C | 0.49242 | 0.40972 | 0.23659 |
| C13 | C | 0.49889 | 0.44180 | 0.21559 |
| N14 | N | 0.54176 | 0.64639 | 0.41138 |
| C15 | C | 0.51749 | 0.65345 | 0.51616 |
| N16 | N | 0.63138 | 0.50794 | 0.51263 |
| C17 | C | 0.66389 | 0.53407 | 0.46122 |
| C18 | C | 0.47741 | 0.31317 | 0.49350 |
| S19 | S | 0.44395 | 0.28097 | 0.27960 |
| C20 | C | 0.45949 | 0.25512 | 0.39199 |
| C21 | C | 0.48703 | 0.27049 | 0.56985 |
| C22 | C | 0.49700 | 0.30428 | 0.62475 |
| C23 | C | 0.50238 | 0.25383 | 0.67696 |
| C24 | C | 0.48894 | 0.22113 | 0.59735 |
| C25 | C | 0.46130 | 0.20576 | 0.42078 |
| C26 | C | 0.44601 | 0.22243 | 0.31316 |
| C27 | C | 0.45132 | 0.17203 | 0.36452 |
| C28 | C | 0.47111 | 0.16316 | 0.49127 |
| S29 | S | 0.50475 | 0.19529 | 0.70394 |
| H30 | H | 0.43167 | 0.43159 | 0.58390 |
| H31 | H | 0.42081 | 0.37645 | 0.60634 |
| H32 | H | 0.50947 | 0.40309 | 0.14583 |
| H33 | H | 0.52183 | 0.45829 | 0.12074 |
| H34 | H | 0.51780 | 0.32048 | 0.75988 |
| H35 | H | 0.52386 | 0.26560 | 0.81602 |
| H36 | H | 0.42461 | 0.21069 | 0.17309 |
| H37 | H | 0.43047 | 0.15590 | 0.22962 |
| H38 | H | 0.62193 | 0.54404 | 0.09179 |
| H39 | H | 0.57246 | 0.54692 | 0.01774 |
| H40 | H | 0.57499 | 0.47426 | 0.79612 |
| H41 | H | 0.52558 | 0.47877 | 0.71954 |
| O42 | O | 0.13911 | 0.50733 | 0.62913 |
| O43 | O | 0.89245 | 0.33146 | 0.39605 |
| H44 | H | 0.56552 | 0.66565 | 0.38945 |
| H45 | H | 0.62882 | 0.48690 | 0.61184 |

**Structural parameters of partially (50%) oxidized Amide-BDT-ETTA**

Unit Cell Parameters (P6):

*a* = *b* = 4.749 nm, *c*= 0.447 nm

*α* = *β* = 90 º, *γ* = 120 º

**Table S3.** Fractional coordinates of partially (50%) oxidized Amide-BDT-ETTA.

| C1 | C | 0.48432 | 148.461 | 0.38418 |
| --- | --- | --- | --- | --- |
| C2 | C | 0.51927 | 0.54774 | 0.38439 |
| C3 | C | 0.54538 | 0.51508 | 0.40932 |
| C4 | C | 0.57251 | 0.53551 | 0.23115 |
| C5 | C | 0.60187 | 0.53578 | 0.26161 |
| C6 | C | 0.60481 | 0.51526 | 0.47096 |
| C7 | C | 0.57813 | 0.49539 | 0.65177 |
| C8 | C | 0.54930 | 0.49634 | 0.62704 |
| C9 | C | 0.45327 | 0.42585 | 0.52296 |
| C10 | C | 0.44866 | 0.39449 | 0.53153 |
| C11 | C | 0.47148 | 0.38751 | 0.41026 |
| C12 | C | 0.49915 | 0.41204 | 0.27012 |
| C13 | C | 0.50448 | 0.44361 | 0.25494 |
| N14 | N | 0.53394 | 0.64495 | 0.43012 |
| C15 | C | 0.50887 | 0.65207 | 0.50951 |
| N16 | N | 0.63381 | 0.51315 | 0.50190 |
| C17 | C | 0.66179 | 0.53232 | 0.37952 |
| C18 | C | 0.48554 | 0.31470 | 0.47330 |
| S19 | S | 0.45191 | 0.28421 | 0.25146 |
| C20 | C | 0.46618 | 0.25691 | 0.35089 |
| C21 | C | 0.49346 | 0.27083 | 0.53285 |
| C22 | C | 0.50438 | 0.30459 | 0.59932 |
| C23 | C | 0.50760 | 0.25287 | 0.63550 |
| C24 | C | 0.49327 | 0.22033 | 0.54624 |
| C25 | C | 0.46604 | 0.20650 | 0.36336 |
| C26 | C | 0.45191 | 0.22443 | 0.26118 |
| C27 | C | 0.45508 | 0.17281 | 0.29547 |
| C28 | C | 0.47361 | 0.16255 | 0.42326 |
| S29 | S | 0.50726 | 0.19275 | 0.64687 |
| H30 | H | 0.43491 | 0.42906 | 0.63342 |
| H31 | H | 0.42727 | 0.37546 | 0.64170 |
| H32 | H | 0.51695 | 0.40680 | 0.17385 |
| H33 | H | 0.52742 | 0.46109 | 0.16548 |
| H34 | H | 0.52517 | 0.31964 | 0.73954 |
| H35 | H | 0.52878 | 0.26368 | 0.77909 |
| H36 | H | 0.43071 | 0.21362 | 0.11768 |
| H37 | H | 0.43443 | 0.15785 | 0.15331 |
| H38 | H | 0.44718 | 0.11295 | 0.24398 |
| H40 | H | 0.62149 | 0.55131 | 0.11336 |
| H41 | H | 0.57082 | 0.55106 | 0.06511 |
| H42 | H | 0.57985 | 0.47951 | 0.81549 |
| H43 | H | 0.52904 | 0.48174 | 0.76732 |
| H44 | H | 0.55792 | 0.66413 | 0.42032 |
| O45 | O | 0.56058 | 0.89147 | 0.58254 |


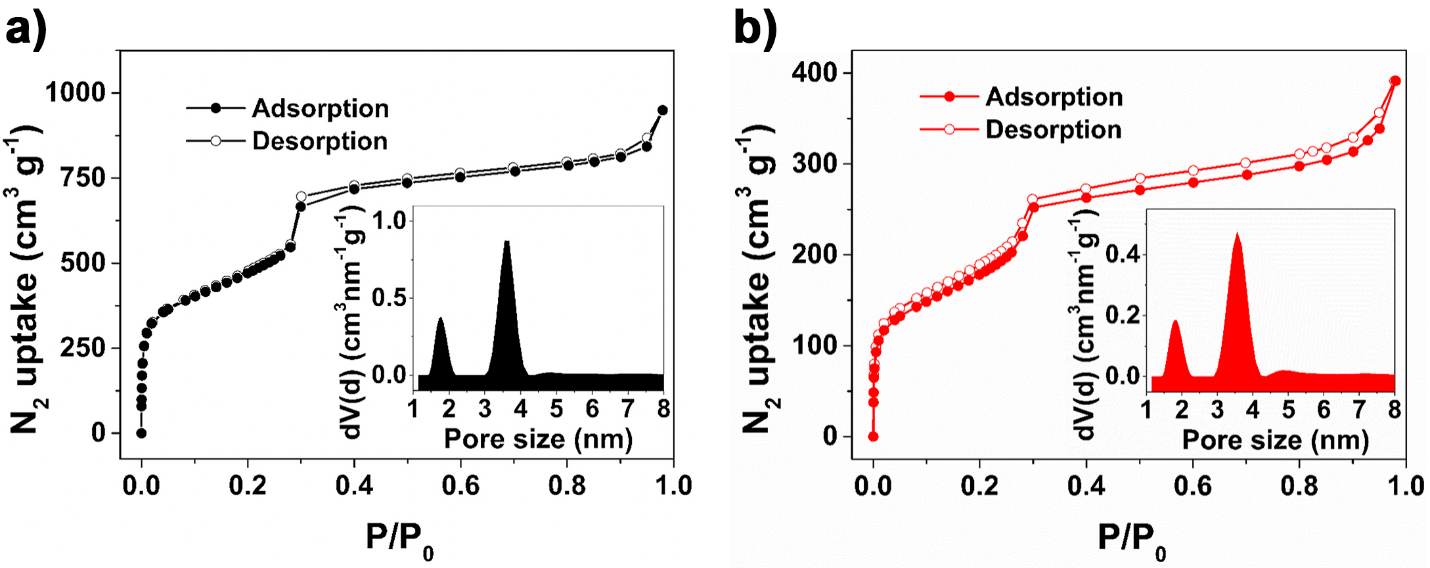


**Figure S3.** Nitrogen sorption isotherms with resulting pore size distribution of (a) Imine-BDT-ETTA and (b) Amide-BDT-ETTA.


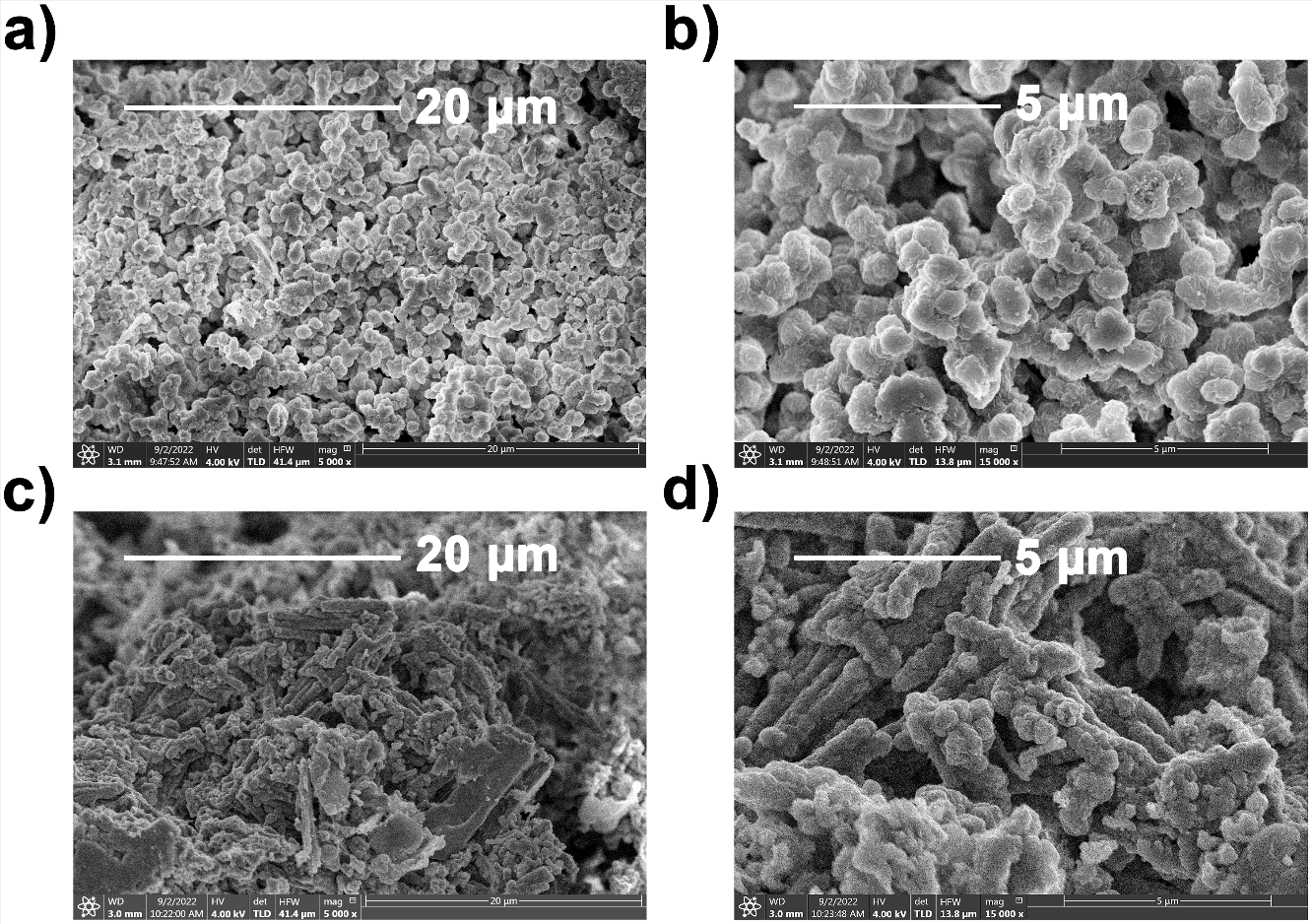


**Figure S4.** SEM images of (a,b) Imine-BDT-ETTA and (c,d) Amide-BDT-ETTA.


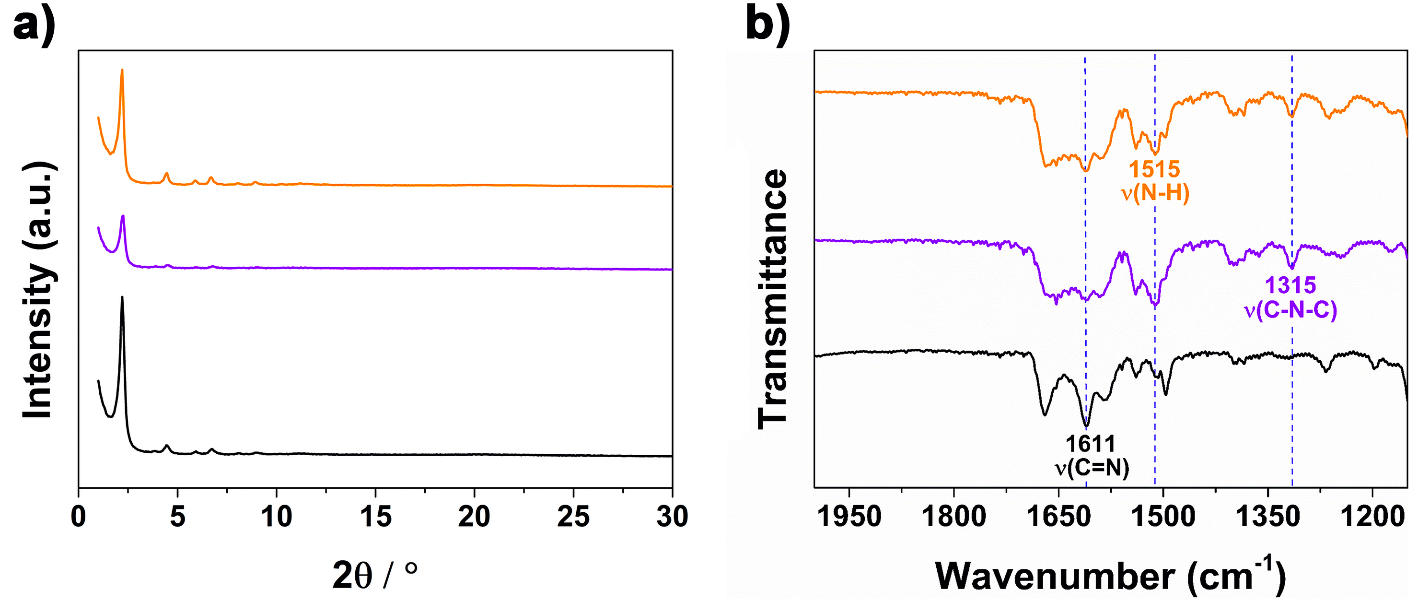


**Figure S5.** (a) PXRD pattern and (b) FT-IR spectra of three reference samples: Reference-COF-1 (black), Reference-COF-2 (purple) and Reference-COF-3 (orange).


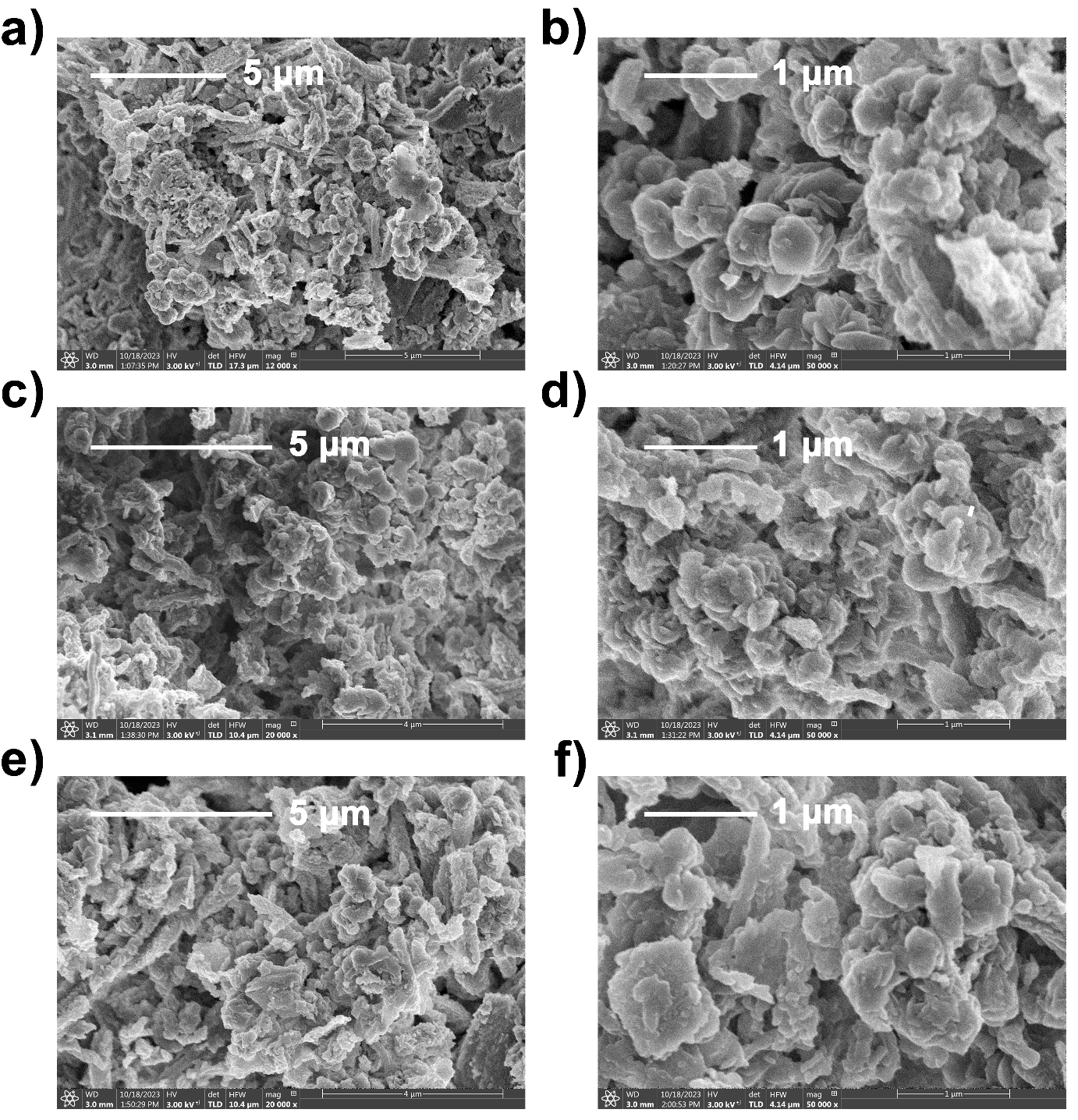


**Figure S6.** SEM images of three reference samples: (a,b) Reference-COF-1, (c,d) Reference-COF-2 and (e,f) Reference-COF-3.


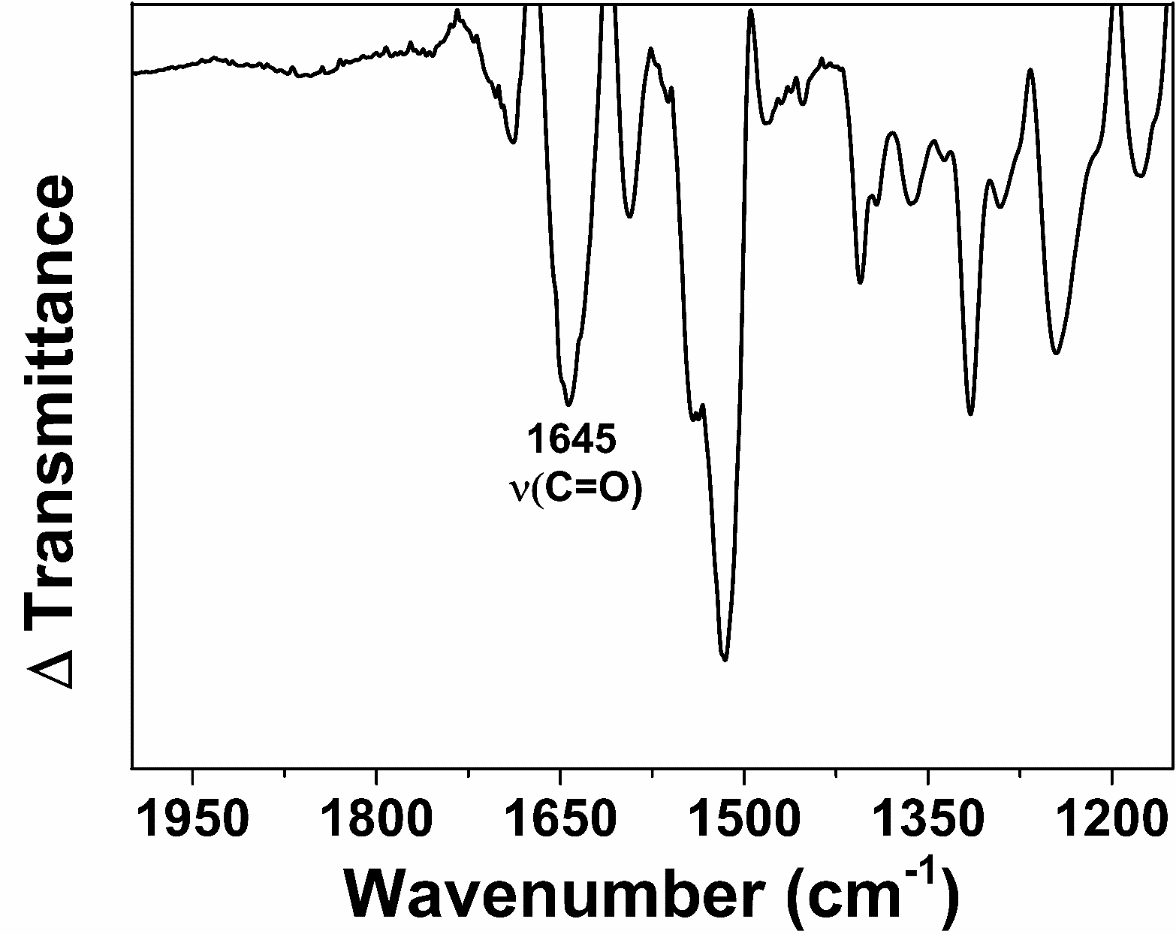


**Figure S7.** FT-IR difference spectrum obtained through the subtraction of the Imine-BDT-ETTA spectrum from Amide-BDT-ETTA spectrum.


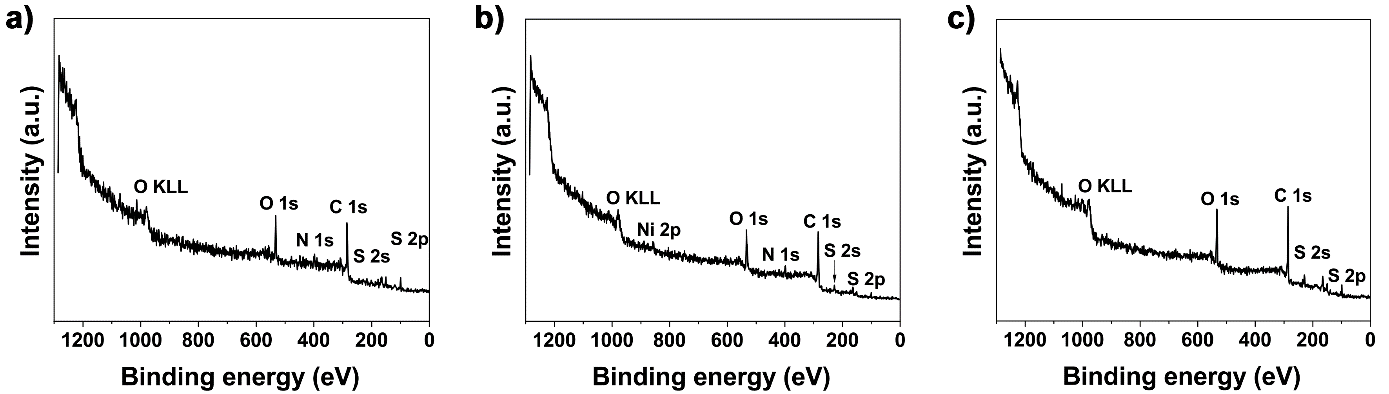


**Figure S8.** Full XPS spectrum of (a) Imine-BDT-ETTA COF, (b) Amide-BDT-ETTA COF and (c) BDT linker. O KLL Auger lines correspond to oxygen Auger transitions with an initial K-shell vacancy and a final double L-shell vacancy. Their kinetic energies remain independent of the ionizing radiation. Ni peak originates from the screws of the sample holder.


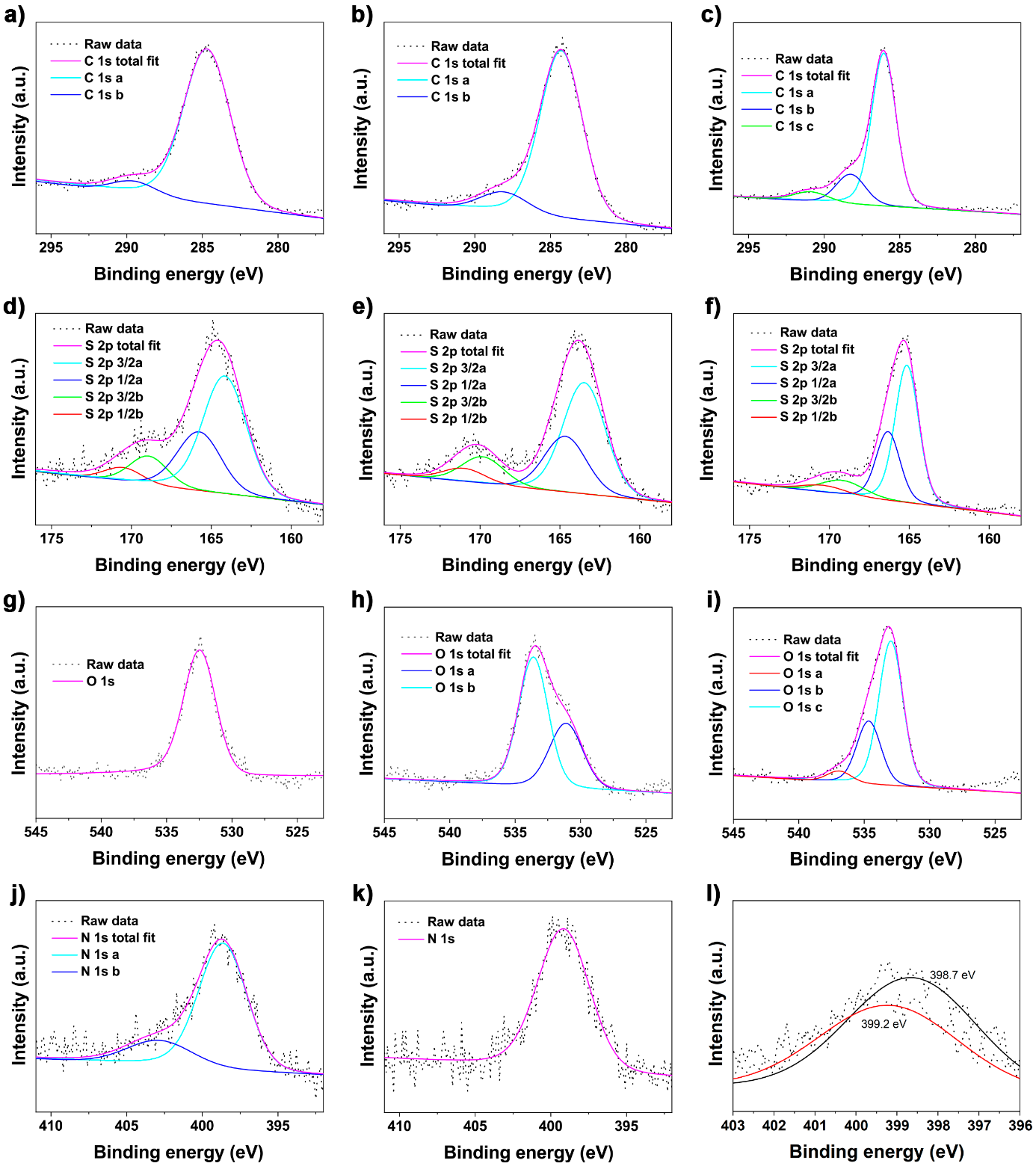


**Figure S9.** Detailed XPS data and analysis. Carbon 1s XPS spectra of (a) Imine-BDT-ETTA, (b) Amide-BDT-ETTA and (c) BDT linker. Two carbon environments are observed for both COFs: at **284.7 eV and 289.5 eV** for Imine-BDT-ETTA, and at **284.3 eV and 288.1 eV** for Amide-BDT-ETTA. The **carbon signals** 284.7 eV and 284.3 eV correspond to overlapping C=C, C–N, and C=N bonds.^[5]^ The **higher binding energy peaks** indicate oxidized carbon species from surface oxidation. The **288.1 eV peak in Amide-BDT-ETTA** is attributed to carbonyl (C=O) moieties,^[6,7]^ while the **289.5 eV peak in Imine-BDT-ETTA** is assigned to unreacted aldehyde groups at surface terminations and lattice defects. S 2p XPS spectra of (d) Imine-BDT-ETTA, (e) Amide-BDT-ETTA and (f) BDT linker. Oxygen 1s XPS spectra of (g) Imine-BDT-ETTA COF, (h) Amide-BDT-ETTA COF and (i) BDT linker. Oxygen species between 532 eV and 536 eV correspond to carbonyl groups, amide bonds and sulfonyl groups.^[8,9]^ Nitrogen 1s XPS spectra of (j) Imine-BDT-ETTA and (k) Amide-BDT-ETTA. Imine-BDT-ETTA revealed two nitrogen species at 398.6 eV (C=N and C–N bonds)^[5]^ and 402.8 eV (ammonium salts).^[10]^ The presence of 402.8 eV signal exclusively in Imine-BDT-ETTA suggests the presence of unreacted (surface-terminating) amino groups remaining after COF formation. For Amide-BDT-ETTA, one nitrogen species was observed at 399.0 eV, which implies in the existence of the C=N and C–N bonds. (l) The comparison between the N 1s spectra of Imine-BDT-ETTA (black) and Amide-BDT-ETTA (red) reveals a shift toward higher binding energy after the amidization process (from 398.7 to 399.2 eV), confirming the efficient conversion of imine to amide.


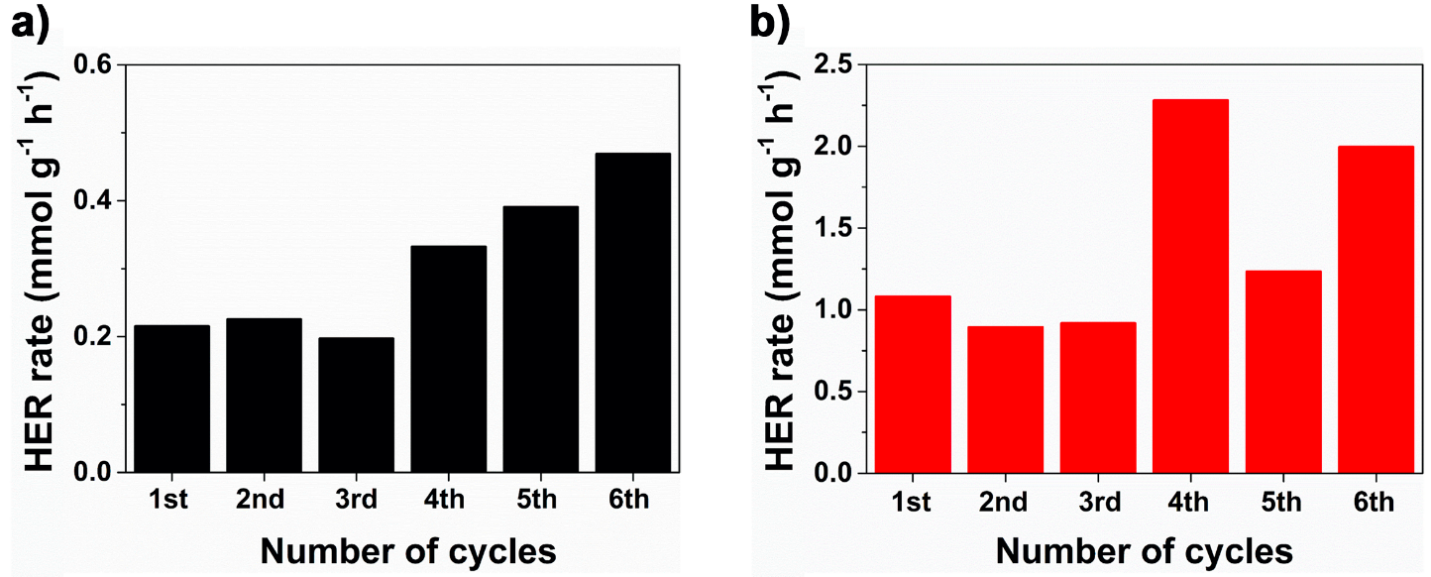


**Figure S10.** HER rates using 10 mM H_2_A (1st-3rd cycles) and 2 mM H_2_A (4th-6th cycles) for (a) Imine-BDT-ETTA and (b) Amide-BDT-ETTA. Conditions of photocatalytic tests: λ > 420 nm, 100 mW cm^-2^, 1 g/L COF suspension in H_2_A, 1.0 wt% (Pt/COF) using H_2_PtCl_6_ precursor.


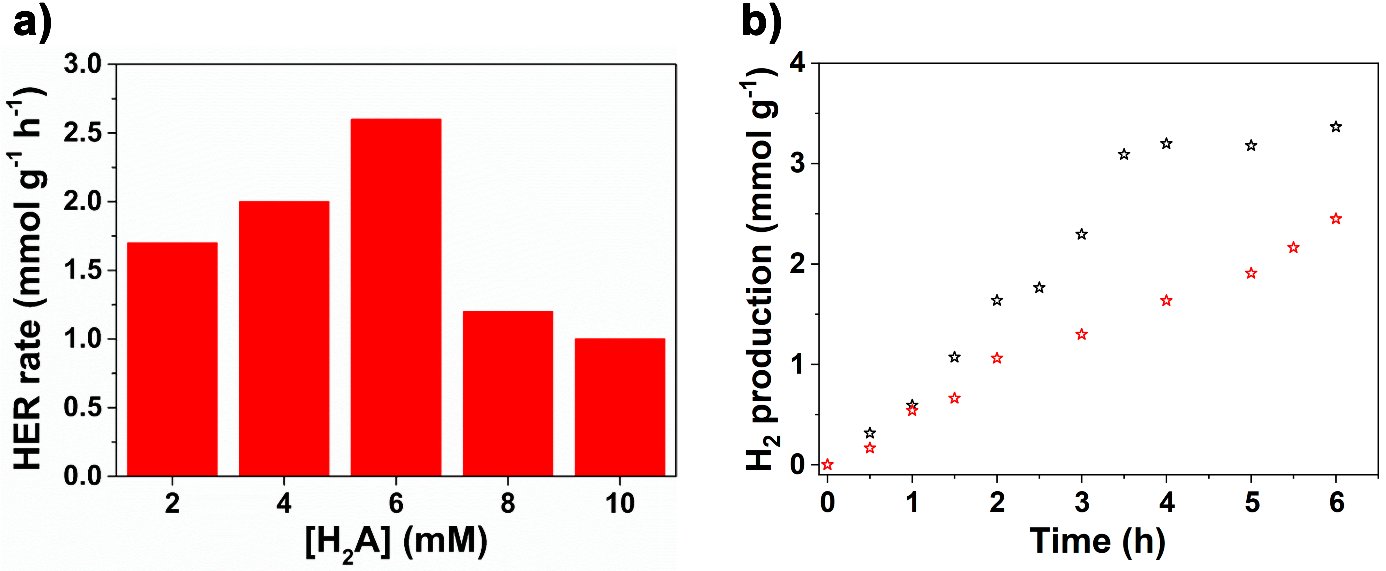
 **Figure S11.** (a) HER rates depending on the concentration of H_2_A for the Amide-BDT-ETTA. (b) H_2_ evolution using 0.75 M TEOA suspension for the Imine-BDT-ETTA (black) and Amide-BDT-ETTA (red), respectively. Conditions of photocatalytic tests: λ > 420 nm, 100 mW cm^-2^, 1 g/L COF suspension, 1.0 wt% (Pt/COF) using H_2_PtCl_6_ precursor.


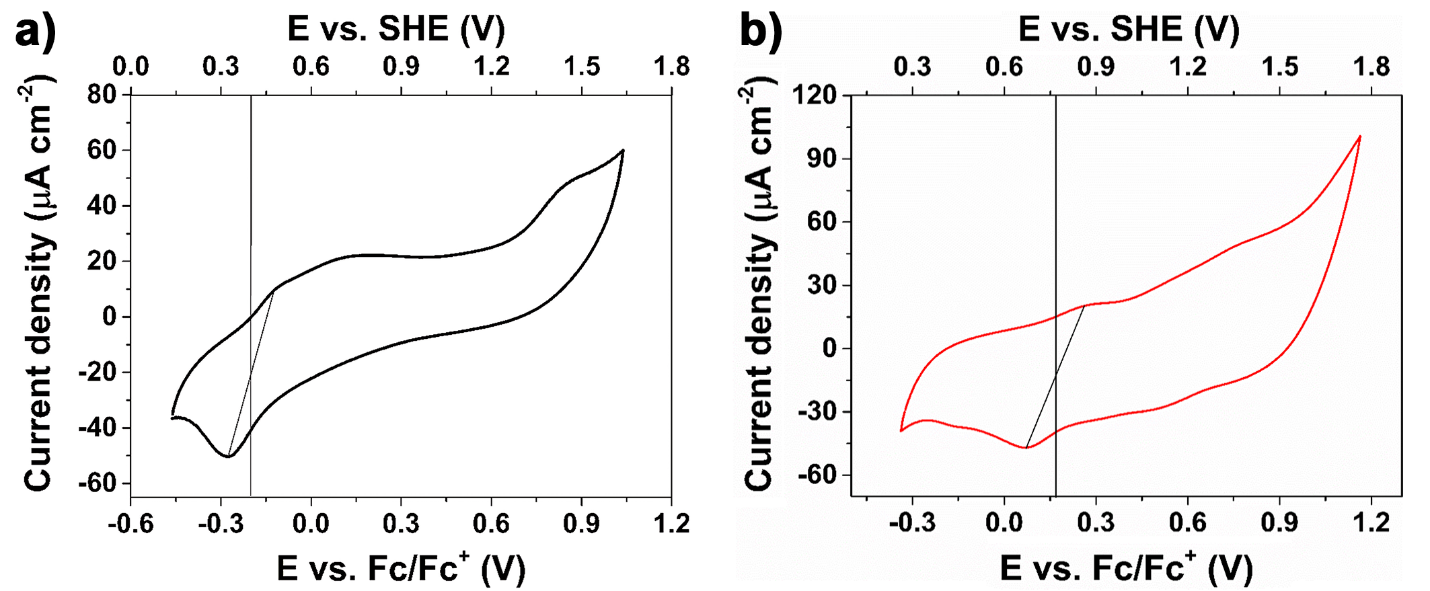


**Figure S12.** CV plot of (a) Imine-BDT-ETTA and of (b) Amide-BDT-ETTA recorded in 0.1 M NBu4PF6 in acetonitrile.


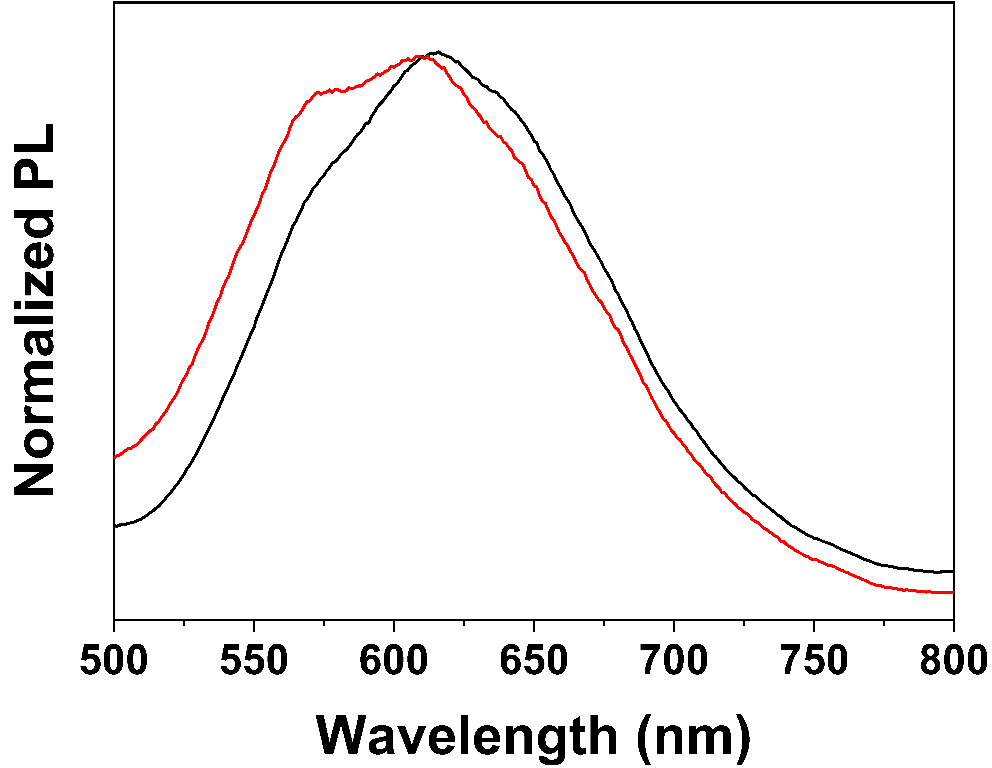


**Figure S13.** PL spectra for Imine-BDT-ETTA (black) and Amide-BDT-ETTA (red).

**Table S4.** pH Values measured at different concentrations of H_2_A.

| **Concentration of H_2_A (mM)** | **pH value** |
| --- | --- |
| 0 | 6.51 |
| 2 | 3.53 |
| 4 | 3.34 |
| 6 | 3.23 |
| 8 | 3.16 |
| 10 | 3.10 |


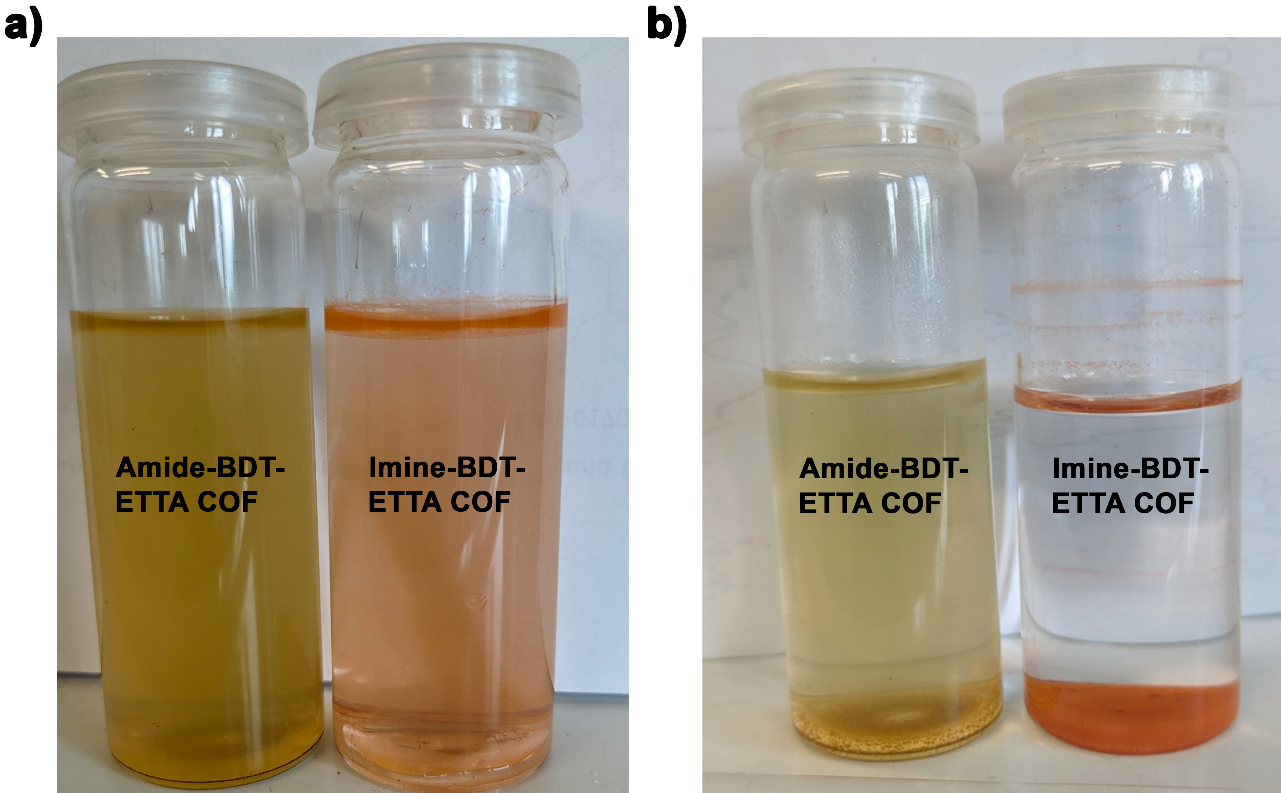


**Figure S14.** Suspensions in water of Amide-BDT-ETTA and Imine-BDT-ETTA (a) after the ultrasonication and (b) one hour after the ultrasonication.


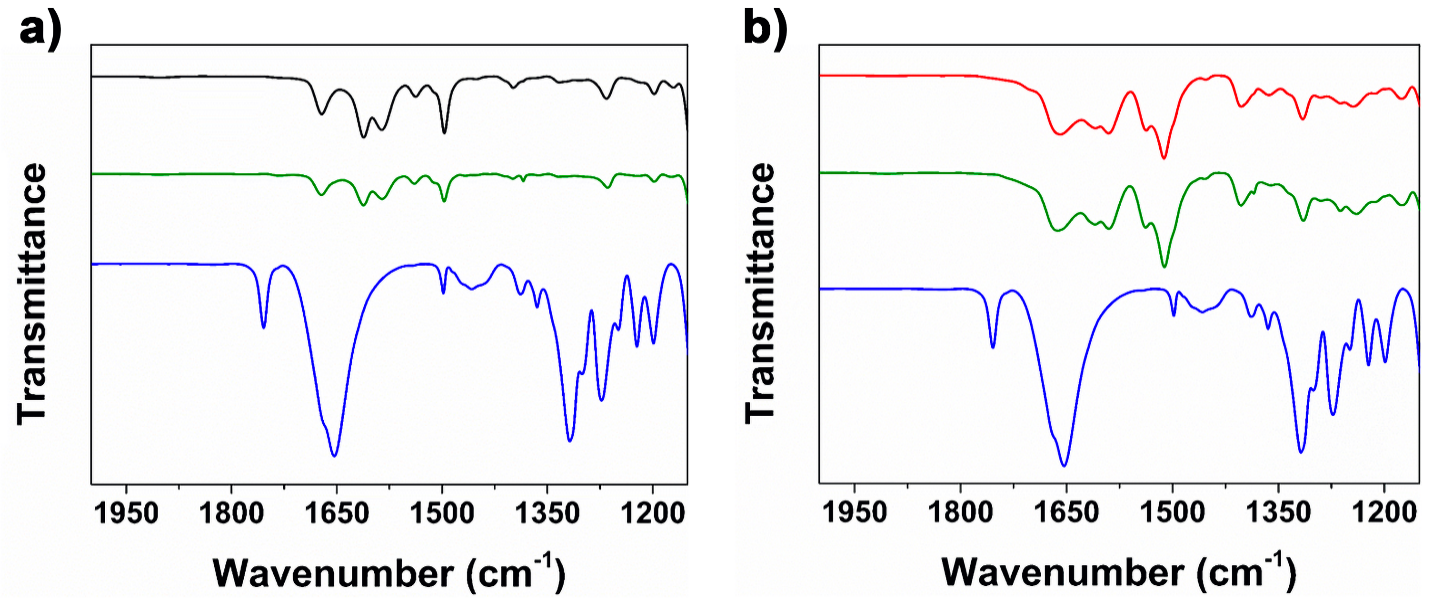
**Figure S15.** FT-IR spectra of (a) Imine-BDT-ETTA (black), Imine-BDT-ETTA treated with H_2_A (green) and H_2_A (blue) and (b) Amide-BDT-ETTA (red), Amide-BDT-ETTA treated with H_2_A (green) and H_2_A (blue).


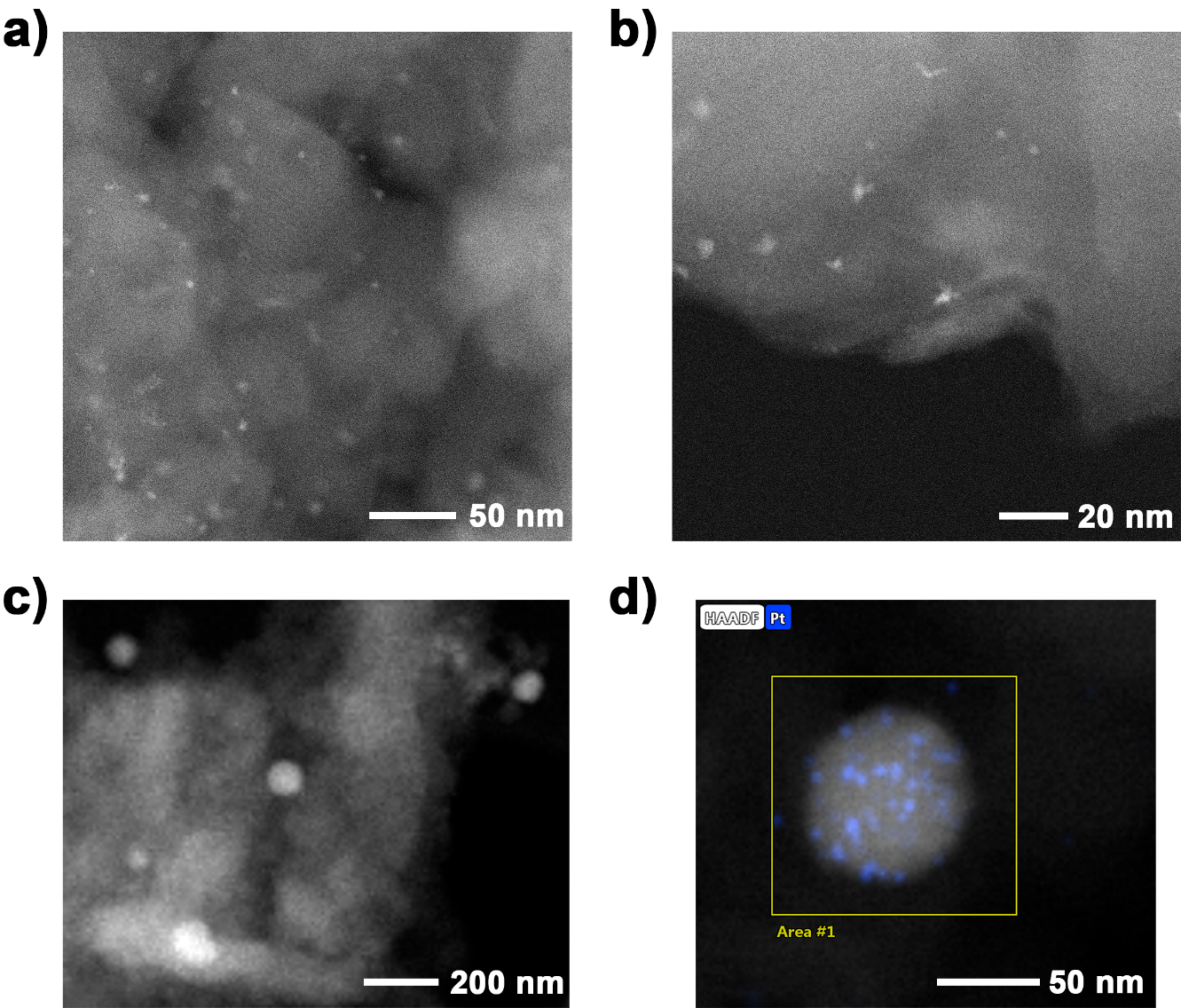


**Figure S16.** Images of Pt particles within (a,b) Imine-BDT-ETTA-COF and (c,d) Amide-BDT-ETTA structure obtained after 13 h illumination. Conditions of illumination: λ > 420 nm, 100 mW cm^-2^, 1 g/L COF suspension containing 10 mM H_2_A, 1.0 wt% (Pt/COF) using H_2_PtCl_6_ precursor.


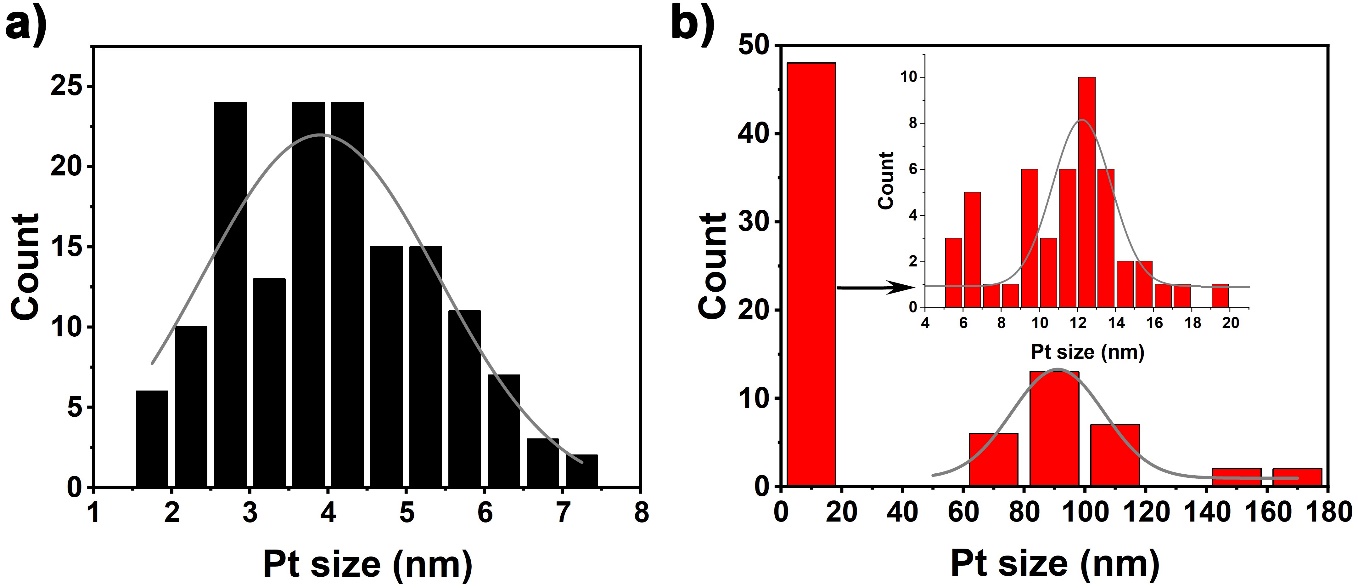


**Figure S17.** Pt size distribution in the (a) Imine-BDT-ETTA-COF and in the (b) Amide-BDT ETTA-COF obtained after 13 h illumination. Conditions of illumination: λ > 420 nm, 100 mW cm^-2^, 1 g/L COF suspension containing 10 mM H_2_A, 1.0 wt% (Pt/COF) using H_2_PtCl_6_ precursor.

**References**

[1] J. M. Rotter, S. Weinberger, J. Kampmann, T. Sick, M. Shalom, T. Bein, D. D. Medina, *Chem. Mater.* **2019**, *31*, 10008.

[2] P. J. Waller, S. J. Lyle, T. M. Osborn Popp, C. S. Diercks, J. A. Reimer, O. M. Yaghi, *J. Am. Chem. Soc.* **2016**, *138*, 15519.

[3] T. Sick, A. G. Hufnagel, J. Kampmann, I. Kondofersky, M. Calik, J. M. Rotter, A. Evans, M. Döblinger, S. Herbert, K. Peters, D. Böhm, P. Knochel, D. D. Medina, D. Fattakhova-Rohlfing, T. Bein, *J. Am. Chem. Soc.* **2018**, *140*, 2085.

[4] G. Greczynski, L. Hultman, *J. Appl. Phys.* **2022**, *132*, 011101.

[5] M. Kehrer, J. Duchoslav, A. Hinterreiter, M. Cobet, A. Mehic, T. Stehrer, D. Stifter, *Plasma Process. Polym.* **2019**, *16*, 1800160.

[6] J. Kettle, Z. Ding, M. Horie, G. C. Smith, *Org. Electron.* **2016**, *39*, 222.

[7] M. A. Leich, N. M. Mackie, K. L. Williams, E. R. Fisher, *Macromolecules* **1998**, *31*, 7618.

[8] G. P. López, D. G. Castner, B. D. Ratner, *Surf. Interface Anal.* **1991**, *17*, 267–272.

[9] B. Folkesson, *Spectrosc. Lett.* **1982**, *15*, 165.

[10] M. Šetka, R. Calavia, L. Vojkůvka, E. Llobet, J. Drbohlavová, S. Vallejos, *Sci. Rep.* **2019**, *9*, 8465.
